# Metabolic engineering of *Saccharomyces cerevisiae* for production of very long chain fatty acid-derived chemicals

**DOI:** 10.1038/ncomms15587

**Published:** 2017-05-26

**Authors:** Tao Yu, Yongjin J. Zhou, Leonie Wenning, Quanli Liu, Anastasia Krivoruchko, Verena Siewers, Jens Nielsen, Florian David

**Affiliations:** 1 Department of Biology and Biological Engineering, Chalmers University of Technology, Kemivägen 10, Gothenburg SE-41296, Sweden; 2 Novo Nordisk Foundation Center for Biosustainability, Chalmers University of Technology, Gothenburg SE-41296, Sweden; 3 Biopetrolia AB, Systems and Synthetic Biology Group, Chalmers University of Technology, Kemivägen 10, Gothenburg SE-41296, Sweden; 4 Novo Nordisk Foundation Center for Biosustainability, Technical University of Denmark, Kongens Lyngby DK-2800, Denmark; 5 Science for Life Laboratory, Royal Institute of Technology, Stockholm SE-17121, Sweden

## Abstract

Production of chemicals and biofuels through microbial fermentation is an economical and sustainable alternative for traditional chemical synthesis. Here we present the construction of a *Saccharomyces cerevisiae* platform strain for high-level production of very-long-chain fatty acid (VLCFA)-derived chemicals. Through rewiring the native fatty acid elongation system and implementing a heterologous *Mycobacteria* FAS I system, we establish an increased biosynthesis of VLCFAs in *S. cerevisiae.* VLCFAs can be selectively modified towards the fatty alcohol docosanol (C_22_H_46_O) by expressing a specific fatty acid reductase. Expression of this enzyme is shown to impair cell growth due to consumption of VLCFA-CoAs. We therefore implement a dynamic control strategy for separating cell growth from docosanol production. We successfully establish high-level and selective docosanol production of 83.5 mg l^−1^ in yeast. This approach will provide a universal strategy towards the production of similar high value chemicals in a more scalable, stable and sustainable manner.

## Abstract (web summary)

Production of chemicals by microbial fermentation is an economical alternative to chemical synthesis. Here the authors re-engineer the yeast *S. cerevisiae* to produce the very long chain fatty alcohol docosanol by expressing a heterologous Mycobacteria fatty acid synthase and a specific fatty acid reductase.

Very-long-chain fatty acids (VLCFAs; 22–26 carbons) are essential biological components, which are incorporated in triacylglycerol molecules, sphingolipids, cuticle or waxes^1^. VLCFAs such as erucic acid, as well as VLCFA-derived products such as very-long-chain fatty alcohols (VLCFAlc) (for example, docosanol) and very-long-chain fatty waxes (for example, Jojoba oil) represent important classes of valuable chemicals, which are widely used as lubricants, detergents, polymers, photographic film-processing agents, coatings, cosmetics and pharmaceuticals^2^,^3^,^4^,^5^.

VLCFA chemicals are mainly extracted from natural sources or synthesized from petrochemical feedstocks. For natural sources, such as vegetable oils, the content of VLCFA derivatives is usually very low and only limited sources are available. When produced from petrochemical feedstocks, VLCFAs are synthesized chemically through oligomerization of ethylene followed by other modifications, which causes production cost increase with chain length^6^. Thus, a more scalable, stable and sustainable production route is needed. One promising solution is microbial production of VLCFAs and their derivatives from renewable feedstocks^7^. Here we engineered the FA metabolism for production of these VLCFA chemicals by the yeast *Saccharomyces cerevisiae*, a robust and industrially established microorganism for sustainable production of several products on the market^8^. As an initial target product we focused on docosanol, a saturated VLCFAlc with a chain length of 22 carbons, which is used as an emollient, emulsifier, thickener in cosmetics and nutritional supplements with a market volume of 40,000 ton per year. Furthermore, docosanol has been approved by the Food and Drug Administration as a pharmaceutical antiviral agent for reducing the duration of cold sores caused by the herpes simplex virus^9^.

Recently, multiple efforts have been initiated on engineering microbial FA synthesis for production of free FAs, alcohols, esters and alkanes^10^,^11^,^12^,^13^,^14^,^15^. Most of these studies have focused on products based on naturally abundant long-chain (LCFAs, 16–18 carbon)^16^, medium-chain (MCFAs, 6–14 carbon) or short-chain (SCFAs, <6 carbon)^17^ FA-derived compounds. The particular chain length can be controlled at many steps during FA synthesis. In yeast, C_16_/C_18_ acyl-CoAs are generated by a type I FA synthase (FAS) system, a multimeric enzyme composed of Fas1 and Fas2 subunits in an α_6_β_6_ complex^16^. Further elongation to VLCFAs is carried out by membrane-embedded enzymes at the endoplasmic reticulum by a four-step process of condensation, reduction, dehydration and reduction^18^. One unit of malonyl-CoA is added after each cycle. In bacteria, FA synthesis is carried out by a type II FAS system, consisting of several separate enzymes. Mycobacteria harbour a special type I FAS I for the biosynthesis of VLCFAs, which are used as building blocks for the synthesis of mycolic acids, major constituents of their cell envelope with a chain length up to 90 carbons. The mycobacterial FAS I is a large multifunctional enzyme, which catalyses the *de novo* synthesis of FAs and shares homology with fungal FAS^19^. It produces both C_16_/C_18_ FAs for the intrinsic cellular demand, as well as C_22_/C_24_/C_26_ FAs for the specific cell wall structures (Supplementary Table 1)^20^.

Unlike LCFA-, MCFA- or SCFA-derived products, total biosynthesis of VLCFA-derived products encounters significant technical challenges: first, the chain length and amount of VLCFAs is controlled by strict cellular regulation mechanisms^21^; second, considering the longer chain length, VLCFAs biosynthesis needs much more reduction equivalents and ATP, which will cause a higher metabolic burden; third, the low concentration of cellular VLCFAs compared with C_16_/C_18_ FAs requires higher activity and specificity of the converting enzyme(s) to generate target products; fourth, the draining of VLCFAs towards the target compound biosynthesis can cause growth defects, as VLCFAs are necessary for essential cellular components such as sphingolipids and nuclear envelope structures^22^. Thus, VLCFA-derived molecule production requires careful tuning and balancing of heterologous metabolic pathways and endogenous FA metabolism.

Here we successfully engineered yeast for the targeted production of VLCFAs and derived products thereof. As a first approach, we selectively modified the endogenous yeast FA elongation system to specifically control the chain length of VLCFAs. In a parallel approach, we demonstrated that the FAS I system from *Mycobacterium vaccae* can be functionally expressed in yeast generating a bimodal product distribution of C_16–18_ and C_22–24_ VLCFAs. Combining these two strategies, we could selectively produce VLCFAs ranging from C_22_ to C_26_ in chain length. Then, we further extended the metabolic pathways by expressing a C_22_-specific VLCFA reductase for the production of docosanol. To relieve the competition for VLCFAs between product formation and cell growth-associated processes, we implemented a dynamic control strategy for dividing the production phase and cell growth phase, which increased the docosanol production almost four-fold. Ultimately, by (re)constructing the whole pathway under this dynamic control, together with further enhancing the yeast elongation system, mycobacterial FAS I system and the precursor supply, we enabled a docosanol production of 83.5 mg l^−1^, an almost 80-fold improvement compared with the initial proof-of-concept strain. Our results show that the FA metabolism can be engineered for the production of VLCFA-derived chemicals in yeast. Furthermore, for the first time the mycobacterial FAS I system was successfully expressed in yeast for controlling the VLCFA chain length, representing a new and feasible strategy for targeted high-level production of VLCFAs and derived products (Fig. 1).

## Results

### VLCFA chain length control by FA elongation system

Normally, yeast generates very low amounts of VLCFAs (≥C_20_). They are derived from the major FA components of C_16_/_18_ LCFAs through several catalytic steps carried out by the intrinsic elongation system. β-Ketoacyl-CoA synthases, also known as elongases, catalyse the first and rate-limiting step of the elongation process by condensing acyl-CoA with malonyl-CoA building blocks^23^. These elongases determine the substrate specificity and final product profile of the whole elongation system^18^. In *S. cerevisiae*, the elongases Elo1, Elo2 and Elo3 are required for VLCFA synthesis. These elongases have different substrate and product specificities: Elo1 has specificity for the elongation of C_12–16_ to C_16–18_ FAs, whereas Elo2 elongates C_16–18_ up to C_22_ and Elo3 elongates C_18_ up to C_26_ (refs 18, 24). We thus modulated these elongase genes for enhancing VLCFAs pools with chain length ≥C_22_.

We first engineered the VLCFAs biosynthesis in the LCFA-CoA overproducing strain JV03, in which the LCFA-CoA-consuming pathways including β-oxidation, triacylglycerol (TAG) and steryl ester synthesis were deleted (CEN.PK 113-5D *are1Δ dga1Δ are2Δ lro1Δ pox1Δ*, Fig. 2a)^25^. This strain JV03 produced a small fraction of C_26_, whereas C_22_ and C_24_ VLCFAs were not detectable (Supplementary Fig. 1a). To drive the FA pool towards C_22_, we deleted the *ELO3* gene to block C_26_ biosynthesis, which led to production of C_22:0_ VLCFAs (strain TY001, Supplementary Figs 1b and 2b). Subsequently, the *ELO2* gene was overexpressed in JV03 *elo3*Δ strain (TY002, Supplementary Table 5), which further increased the C_22_ VLCFA level by almost 1.5-fold (Fig. 2b). On the other hand, when overexpressing the *ELO3* gene in the JV03 background strain TY003, the C_26_ VLCFA level increased almost twofold (Supplementary Fig. 1d). These results clearly showed that the chain length of VLCFAs could be selectively modulated by engineering the intrinsic yeast elongation system. Despite using an engineered strain, the overall production of C22 FAs was lower than 1 mg g^−1^ dry cell weight (DCW).

### VLCFAs chain length control by *Mycobacterium* FAS I system

To overcome the limitation of the intrinsic yeast elongation system, we focused on implementing the mycobacterial FAS system FAS I into yeast. In mycobacteria, this multimeric enzyme produces C_16_/C_18_ FAs for the intrinsic cellular demand, as well as C_22_/C_24_/C_26_ FA precursors for specific mycobacterial cell wall structures^20^,^26^. We attempted to establish an additional source for VLCFAs production in yeast by expressing the *M. vaccae* FAS I gene *(mvfas)*, which can specifically generate C_22_/C_24_, as well as C_16_/C_18_ FAs. The MvFAS was selected as it provides high levels of C_22_ VLCFAs as direct precursor for docosanol production. Compared with the multi-domain yeast Fas1/2 system, the MvFAS lacks the phosphopantetheinyl transferase domain and thus needs a separate acyl carrier protein synthase (Acps) to activate the ACP with phosphopantetheine. To guarantee a functional MvFAS, a cognate *acps* gene from *M. vaccae* was expressed in fusion with the carboxy-terminal *mvfas* gene via a 3*GGGGS linker. This chimeric MvFAS-AcpS successfully restored the growth of a FA auxotrophic yeast strain PWY12 (MATα *ura3Δ leu2Δ his3Δ trp1Δ can1Δ fas1Δ::HIS3 fas2Δ::LEU2)*^27^ on yeast extract peptone dextrose medium (YPD) or complete supplement mixture (CSM) synthetic media without FA addition (Supplementary Fig. 2). This result demonstrated that *mvfas-acps* was functionally expressed in *S. cerevisiae.* FA profile analysis showed that *mvfas-acps* expression in strain TY005 enabled a threefold increase in the C_26_ VLCFA production level but only small amounts of C_22_ or C_24_ VLCFAs were produced (Supplementary Fig. 3). The high level of C_26_ VLCFA may be caused by the function of endogenous yeast elongation system through further elongation of the C_22_ and C_24_ FAs produced by the MvFAS. To avoid the interference, we expressed the *mvfas-acps* gene in an *elo2Δ/elo3Δ* double knockout strain^28^, which is growth deficient even with VLCFAs supplementation. Thus, an *ELO3* expressing plasmid pELO3 was introduced to this strain for complementing the growth deficiency (strain TDY7005). To test the mycobacterial FAS system in this background strain, the plasmid p415GPD-MvFAS-AcpS was introduced into strain TDY7005. These transformants were screened for growth on 5-fluoroorotic acid (5-FOA) plates to obtain the strain TY004, which had lost the plasmid carrying the *ELO3* gene (Fig. 2c). The results showed that *mvfas-acps* is able to replace the *ELO3* gene in the *elo2Δ/elo3Δ* background strain. Total FAs analysis of the strain TDY7005 showed that this strain also generated C_26_ VLCFAs due to the overexpression of *ELO3* (Supplementary Fig. 4b), which is consistent with previous results (Supplementary Fig. 1c). However, the strain TY004, expressing a functional version of *mvfas-acps*, produced high levels of C_22_ and C_24_ FAs as expected from the native activity of the enzyme (Fig. 2c and Supplementary Fig. 4a).

These results show that the mycobacterial FAS I system is able to replace both the FA synthesis (*FAS1/FAS2*) and elongation (*ELO2*/*ELO3*) function in yeast. By eliminating intrinsic competing elongation reactions, we were able to selectively produce VLCFAs with chain length of C_22_ and C_24_. Expressing MvFAS led to almost fourfold higher production of C_22_ VLCFAs compared with engineering of the endogenous yeast elongation system (Fig. 2b,c). The increased production is likely to be the result of two factors: (1) MvFAS can generate C_16_ and C_18_ by itself and also can elongate the C_16_/C_18_ acyl-CoAs produced by the yeast FAS and (2) using the exogenous MvFAS circumvents the intrinsic regulation mechanisms. Thus, the mycobacterial FAS I system allows for efficient production of specific VLCFAs. In the future, other VLCFAs could be produced via selectively choosing the particular mycobacterial FAS system that corresponds to the VLCFA chain length desired, as summarized in Supplementary Table 1.

### Selection of FA reductase for docosanol production

After establishing the VLCFAs biosynthesis platform, we aimed to engineer it towards docosanol production, a high-value VLCFA-derived chemical (Fig. 3a). For this purpose, two main challenges have to be faced: first, the low level of VLCFAs asks for efficient ‘terminal enzyme(s)’ to convert VLCFAs towards relevant chemicals. Second, with much higher levels of cellular C_16_ and C_18_ fatty acyl-CoAs, the terminal enzyme(s) should have high catalytic specificity. For docosanol production, we tested five fatty acyl-CoA reductases that are capable of specifically converting VLCFA-CoAs towards VLCFAlcs (AmFAR from *Apis mellifera*^29^, AtFAR from *Arabidopsis thaliana*^30^, CfFAR from *Calanus finmarchicus*^29^, ScFAR from *Simmondsia chinensis*^30^ and TaFAR from *Triticum aestivum*^31^, detailed description in Supplementary Table 8). We compared their activity in the strains with increased C_22_-CoA pools. TY001 (JV03 *elo3*Δ) and TY002 (JV03 *elo3*Δ pELO2) were transformed with the respective plasmids resulting in strains TY006–TY015 (Supplementary Table 2). The fatty alcohol profile analysis revealed that most of the reductases tested did not show any detectable docosanol production, probably due to low conversion efficiency and specificity (Supplementary Table 8). Only *atfar* expression in strain TY002 led to the production of detectable amounts of 1.1 mg l^−1^ docosanol (Fig. 3b and Supplementary Table 2). Interestingly, no viable transformants were obtained when attempting to introduce *atfar* expressing plasmid to the strain TY001 (JV03 *elo3*Δ) (without *ELO2* overexpression) (Supplementary Table 2). This might be attributed to the fact that AtFAR-deprived essential C_22_-CoA towards docosanol biosynthesis. *ELO2* overexpression in strain TY012 (JV03 *elo3*Δ pVLCFAlc07) restored the cell growth possibly by providing higher levels of VLCFAs (Fig. 2b) for both docosanol biosynthesis and cell growth. Still, the biomass yield was much lower (final OD_600_=0.3) than for the control strain without expressing *atfar* (final OD_600_=6.0) (Fig. 3c). These results suggested that the docosanol biosynthesis deprived the cell of VLCFAs for cell growth. The metabolic flux therefore should be carefully balanced, to enable better cell proliferation and higher docosanol production. Furthermore, a*tfar* expression in yeast resulted in specific production of docosanol (Fig. 3d,e), proving the high selectivity of this enzyme. This specificity was also shown previously in *A. thaliana* where a mutant of the *atfar* gene reduced the amount of C_22:0_–OH in the suberin isolated from roots and sead coats by 30%, whereas other fatty alcohols were not affected^32^.

### Dynamic control system to increase docosanol production

We successfully demonstrated the concept of docosanol production in yeast (Figs 3b and 4a). However, the titres remained very low due to the impaired cell growth caused by deprivation of essential precursors C_22_-CoA. Thus, we needed to fine tune the expression of these genes to balance cell growth and product formation. To reach this goal, we designed a dynamic control system where cell growth and docosanol production were separated into two different phases by using carbon source-dependent promoters (Fig. 4b and Supplementary Fig. 8). To make the system as stringent and robust as possible, we tested the strategies in JV03 *elo3*Δ strain. As a first attempt to increase cell growth with *atfar* expression, we expressed *ELO3* under the control of *HXT1* promoter, which is activated by high level of glucose and becomes repressed as the level of glucose decreases^33^. For *atfar* expression, we selected *HXT7*, *ADH2*, *ICL1* and *GAL1* promoters^33^, which are repressed by high level of glucose and activated when the cells are in the ethanol phase or exposed to galactose as inducer, respectively (Fig. 4b). In addition, to evaluate constitutive expression of the *atfar* gene, we also tested the *TDH3* and *TEF1* promoters. The results of this screening are summarized in Supplementary Table 3 and highlighted in Fig. 4c (strains TY016-TY027). The strain TY018, having a deletion of *ELO3* and *atfar* expressed under *GAL1* promoter control, showed the highest docosanol production of 4.2 mg l^−1^, which is fourfold higher compared with the previous proof-of-concept strain TY012 (Figs 3b and 4c). The biomass yield was almost 80% of the control strain TY001 (without *atfar* expression). Further expression of *ELO3* under *HXT1* promoter control completely restored the cell growth, but docosanol biosynthesis was totally abolished. In the strains carrying *HXT1*p-*ELO3*, tuning the *atfar* expression with promoters *HXT7*p or *ADH2*p resulted in docosanol production of 0.8 and 0.2 mg l^−1^, respectively, which was much lower than the TY018 strain. These results indicated that *ELO3* disruption is essential for supplying C_22_-CoA for docosanol production and a strict growth phase-dependent control of *atfar* is necessary for fine tuning cell growth and increasing final docosanol titres. In conclusion, the dynamic control of gene expression successfully solved the growth defect caused by precursor deprivation which laid the foundation for high-level production of docosanol.

### Efficient pathway reconstruction for production of docosanol

Although FA metabolism was successfully engineered for docosanol production, the titre needs to be further improved. Owing to the non-functional FA storage and degradation, the growth rate and final cell biomass of the JV03 background strain are lower than that of wild-type strain^25^. We thus re-engineered FA elongation and reduction towards docosanol production in the more robust background strain CEN.PK113-5D. With the recently established CRISPR/Cas9 technology^34^,^35^ and modular pathway engineering^36^ for yeast genetic engineering, we could easily transfer our metabolic engineering strategy to different genetic background strains. Here we mainly focused on integrating the genes into chromosome instead of using episomal expression to get more stable genotypes^37^. We reconstructed the entire pathway for docosanol production including modifications regarding chain-length control, terminal FAR enzyme expression and further optimization using the dynamic control system (Fig. 5a). First, we deleted *ELO3* gene and integrated the *atfar* gene under *GAL1* promoter control at the site of *ELO3* simultaneously; the resulting strain TY028 produced 0.65 mg l^−1^ docosanol. Further overexpressing *ELO2* gene under the control of *GAL10* promoter (strain TY029) improved the docosanol production by 4-fold (2.4 mg l^−1^) compared with the previous strain TY028. To provide more acyl-CoA precursors for the endoplasmic reticulum-localized yeast elongation system, we overexpressed *ELO1* gene under *GAL7* promoter (strain TY030, Supplementary Fig. 9). However, this modification had only a marginal effect on docosanol production (Fig. 5b), suggesting that precursor supply for the elongation system in terms of acyl-CoAs seemed not to be the rate limiting step at this point. In parallel, we aimed to further increase FA production by integrating and overexpressing a constitutive active version of the ACC1** enzyme (*Acc1*^S659A,S1157A^) in strain TY028, thereby leading to an increased precursor supply of malonyl-CoA^1^,^38^. At the same time, we implemented the deletion of the *GAL1* gene to avoid potential inducer consumption and ensure stable *GAL* promoter performance^39^. The resulting strain TY033 could generate 1.4 mg l^−1^ docosanol, more than a twofold increase compared with the previous strain TY028. If further combined with the overexpression of *ELO2* and *ELO1*, the docosanol production was remarkably increased to 5.7 mg l^−1^ (strain TY034) and 51 mg l^−1^ (strain TY035), respectively (Fig. 5b). This highlights that upregulating the elongation system, especially *ELO1*, only becomes beneficial when sufficient malonyl-CoA precursors for FA production are provided. By implementing the MvFAS system, the production level of docosanol was further increased. TY036, which provided increased malonyl-CoA precursor supply through ACC1** overexpression, generated docosanol up to 40 mg l^−1^. When combining both the yeast elongation system and the MvFAS strategy in strain TY037, the level of docosanol was increased to 83.5 mg l^−1^, an almost 80-fold increase compared with the proof-of-concept strain TY012 (JV03 *elo3*Δ pVLCFAlc07) (Figs 3b and 5b). Even after 80-fold improvement in production, docosanol is still the major fatty alcohol peak (Supplementary Fig. 5), demonstrating the high potential and selectivity of the implemented docosanol production pathway.

## Discussion

VLCFAs and their derivatives are important precursors for valuable chemicals used in the food, drug, chemical and cosmetic industry. Here we established a yeast-based production platform for the production of high-value chemical docosanol. This was accomplished by overexpressing a highly active and specific FAR enzyme to generate VLCFAlcs and by controlling FA chain length towards VLCFAs.

During the last years there have been several reports on engineering FA metabolism for long-chain FA-derived chemical and biofuel production^10^,^11^,^40^,^41^,^42^ with the main focus on introducing heterologous pathways to transform cellular abundant FAs (mainly C_16_/_18_). Here we succeeded to engineer FA metabolism towards VLCFA-derived chemical production. Unlike abundant C_16_/_18_ FAs, the VLCFAs biosynthesis is strictly regulated, which makes it challenging to redirect the cellular metabolism towards the production of VLCFA-derived chemicals with specific chain length, such as docosanol. In our study, single modification of VLCFA biosynthesis using the mycobacterial MvFAS resulted in an accumulation of only C_26_ FA, but no C_22_ and C_24_ FAs (Supplementary Fig. 3). This suggests that the cellular elongation system is highly interconnected with the heterologous FAS. Thus, for the production of specific VLCFA chemicals, the intrinsic elongation system should be carefully tuned, depending on the target product of interest. Here we found that disruption of *ELO3* and overexpression of *ELO1* and *ELO2* are the best combination for increased docosanol production. However, engineering the elongation system only produced small amounts of docosanol (1.1 mg l^−1^, Fig. 3b). By additionally introducing the MvFAS system, as well as enhancing the precursor supply, we significantly improved docosanol production by 80-fold to 83.5 mg l^−1^ (Fig. 5b). A heterologous mycobacterial FAS I system was successfully constructed by fusing the phosphopantetheinyl transferase domain with the main FAS enzyme and was proven to be functional and efficient for VLCFA biosynthesis, and to enable high-level docosanol production in yeast (Fig. 5b).

Another challenge for producing VLCFA-derived chemicals is the need for a ‘terminal enzyme’ to selectively transform the precursor VLCFA-CoA into a relevant product, as VLCFA-CoA levels are much lower compared with C_16_/C_18_-CoAs. Thus, an efficient and specific FAR was selected, enabling the high level and targeted production of docosanol (Fig. 3e and Supplementary Fig. 5b). However, an efficient FAR might drain VLCFA-CoA precursors towards the end product, resulting in insufficient VLCFAs supply for cell growth. Correspondingly, we observed that constitutive expression of *atfar* decreased the biomass yield significantly (Fig. 3c). This is counterproductive as a certain amount of biomass is necessary to enable high-level production of the product of interest. The problem was solved by fine-tuning target gene expression using galactose-inducible promoters, thereby dynamically controlling the product pathway and separating the cell growth from the docosanol production phase. Targeted pathway overexpression and control via *GAL* promoters were previously used to optimize artemisinin production. The strategy was applied to avoid build-up of any potentially toxic intermediates in the mevalonate pathway, which may have resulted in strain instability^39^. Furthermore, in our study the strains benefited from this effect. When implementing the constitutively active version of the Acc1 enzyme, high levels of precursor are generated but at the same time this strain shows high instability^10^, possibly due to significant deprivation of the acetyl-CoA pool. To further improve docosanol production in the future, different strategies for increasing cytosolic acetyl-CoA supply could be evaluated^43^.

Recently, several studies showed the reconstruction of fatty acyl-CoA^16^,^44^ or FA^10^ reduction pathways in *S. cerevisiae*, which enabled the production of about 100–300 mg l^−1^ fatty alcohols with a chain-length mixture of C_10_–C_18_. Although facing additional challenges when producing VLCFAs and derived products, we reached a similar range in our studies with a final docosanol titre of 83.5 mg l^−1^ with high purity.

**Figure 1:**
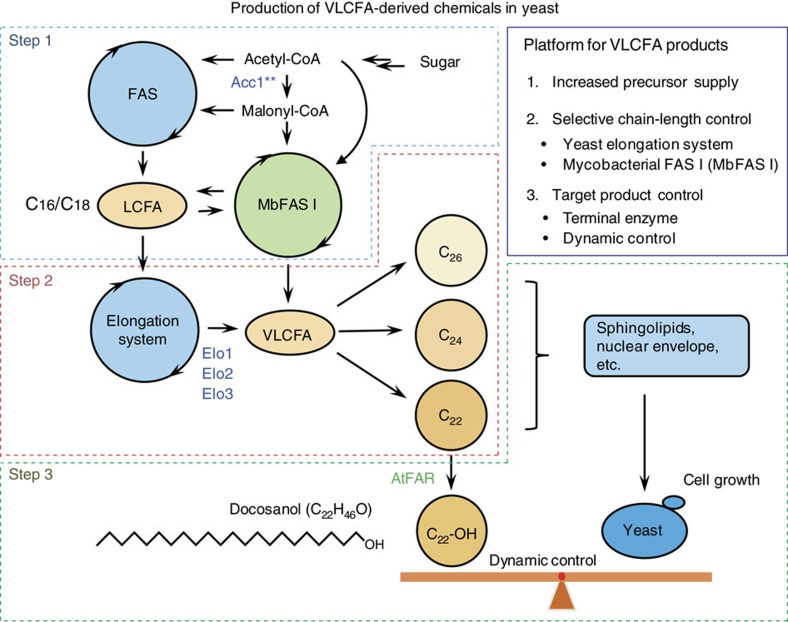
Schematic overview describing platform technologies and toolboxes for targeted VLCFA and derived chemical production in yeast. LCFAs are specifically elongated to a VLCFA of choice via the intrinsic elongation system or Mycobacteria FAS I (MbFAS I) and further modified to the target product of interest. Product selectivity is determined by choice of enzymes and background strain.

**Figure 2:**
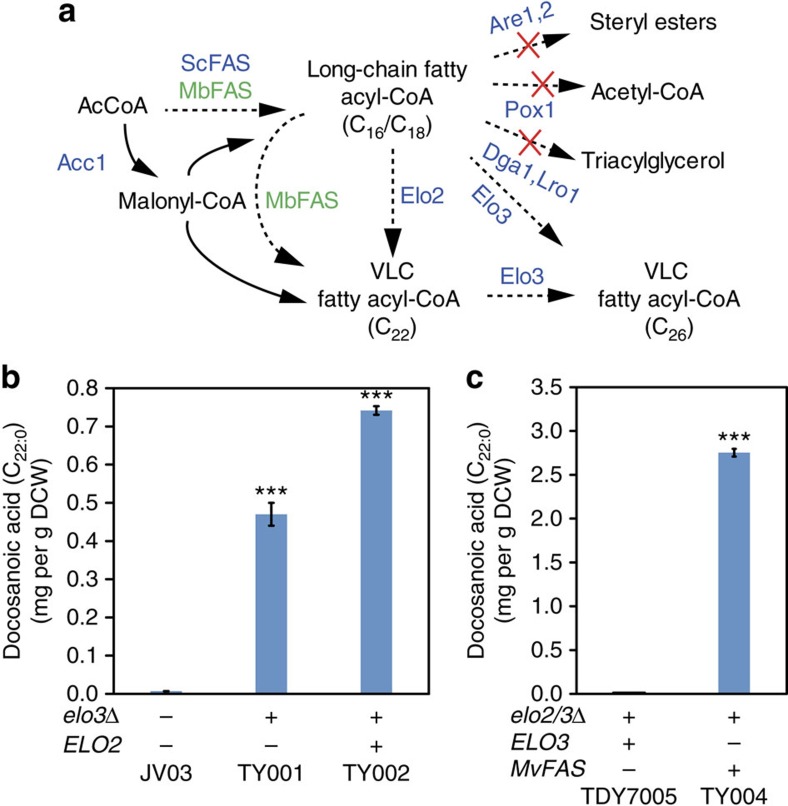
Engineering production of C_22_ VLCFAs by FA chain-length control in yeast. (**a**) Schematic overview of two strategies for chain length control towards VLCFAs. The dotted lines indicate multiple steps and solid lines a single step. Overexpressed genes are shown in blue (endogenous) or green (heterologous). Competitive pathways were eliminated by deleting corresponding genes (marked with X). Elo1, Elo2 and Elo3, yeast FA elongases 1, 2 and 3; MbFAS, FAS I system from *Mycobacterium*; ScFAS, *S. cerevisiae* FAS. (**b**) GC–MS analysis of docosanoic acid (C_22:0_ FAME) generated by yeast elongation system in strains TY001 (JV03 *elo3*Δ) and TY002 (JV03 *elo3*Δ pELO2). Statistical analysis was performed using a Student’s *t*-test (one-tailed; *P<0.05; **P<0.01 and ***P<0.001; two-sample unequal variance). At least two independent measurements were performed for each experiment and the mean±s.d. of three biological replicates of a representative measurement is shown. (**c**) GC–MS analysis of docosanoic acid generated in strain TDY7005 (*MAT*a *lys2 ura3–52 trp1*Δ *leu2*Δ *elo2*Δ::*kanMX elo3*Δ::*TRP1*/pELO3) and strain TY004 (*MAT*a *lys2 ura3–52 trp1*Δ *leu2*Δ *elo2*Δ::*kanMX elo3*Δ::*TRP1*/pGPD415-MvFAS-AcpS). Statistical analysis was performed using a Student’s *t*-test (one-tailed; *P<0.05, **P<0.01 and ***P<0.001; two-sample unequal variance). At least two independent measurements were performed for each experiment and the mean±s.d. of three biological replicates of a representative measurement is shown. All cells were grown as described in experimental procedures.

**Figure 3:**
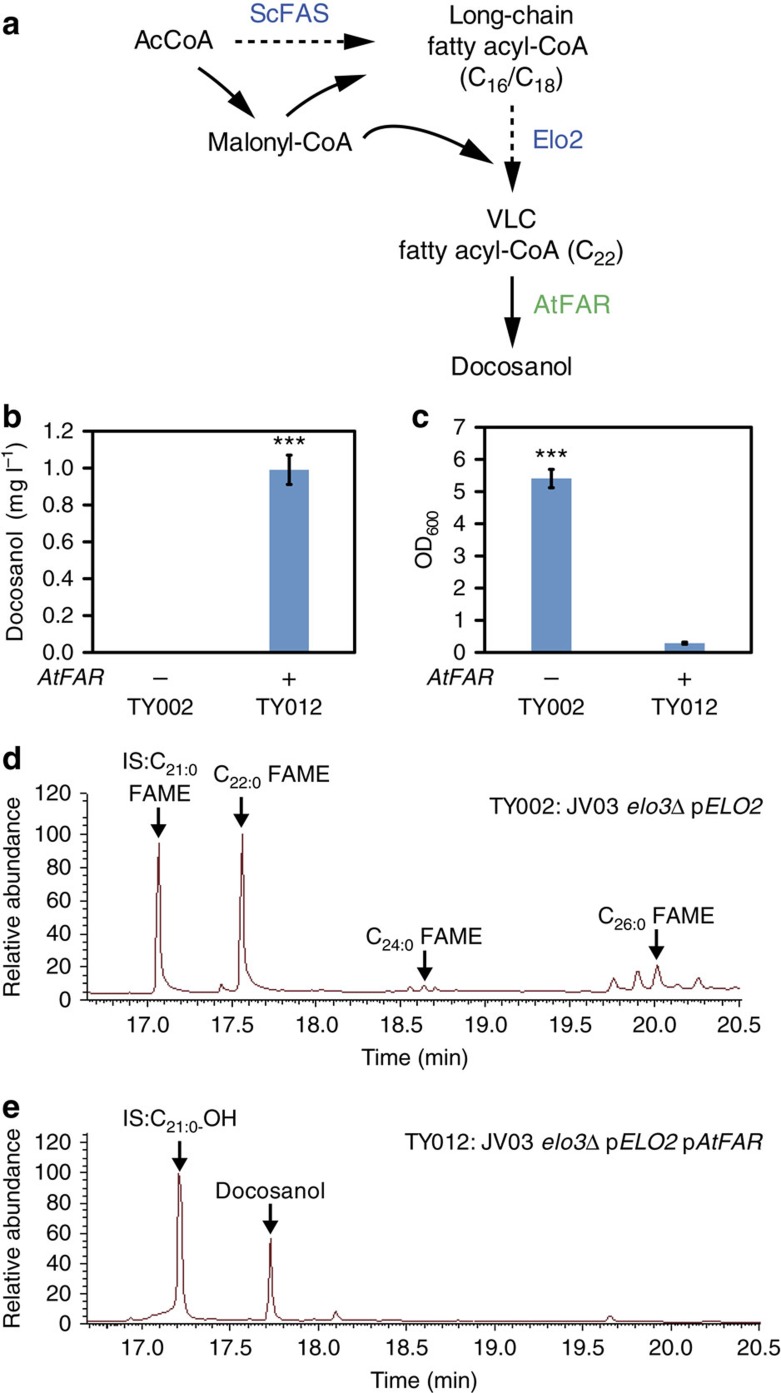
Engineering production of docosanol (C_22_H_46_O) in yeast. (**a**) Schematic biosynthetic pathway for docosanol. AtFAR, fatty acyl-CoA reductase from *A. thaliana* (AT5g22500). (**b**) GC–MS analysis of docosanol production in TY002 (JV03 *elo3*Δ pELO2) with or without expression of *atfar.* Statistical analysis was performed using a Student’s *t*-test (one-tailed; *P<0.05, **P<0.01 and ***P<0.001; two-sample unequal variance). At least two independent measurements were performed for each experiment and the mean±s.d. of three biological replicates of a representative measurement is shown. (**c**) Final OD_600_ of strains TY002 and TY012, with or without expression of the fatty acyl-CoA reductase gene *atfar*. Statistical analysis was performed using a Student’s *t*-test (one-tailed; *P<0.05, **P<0.01 and ***P<0.001; two-sample unequal variance). At least two independent measurements were performed for each experiment and the mean±s.d. of three biological replicates of a representative measurement is shown. (**d**) VLCFA chain-length profiles of strain TY002 (JV03 *elo3*Δ pELO2). IS (internal standard). (**e**) VLCFAlc profiles of docosanol producing strain TY012 (JV03 *elo3*Δ pVLCFAlc07). Typical GC–MS total ion chromatograms (TIC) of total FAs or fatty alcohols extracted from strain TY002 (retention time from 16 to 21 min) and strain TY012 (retention time from 16 to 21 min). FAME and fatty alcohols were identified by retention time and comparison with the mass spectral library. All cells were grown as described under experimental procedures.

**Figure 4:**
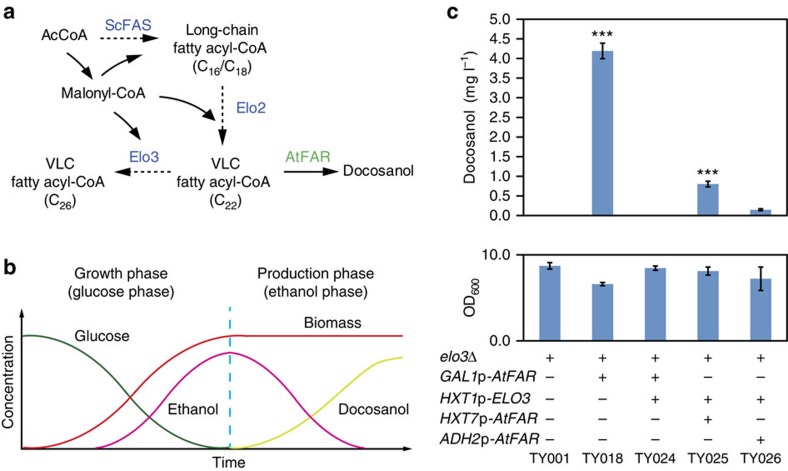
Dynamic control of gene expression for increasing docosanol production. (**a**) Schematic pathway describing the reason for growth defect. (**b**) Schematic illustration describing the dynamic control system, where cell growth and docosanol production are separated into two different phases using carbon source dependent promoters. (**c**) Docosanol production and final culture OD_600_ in relevant strains. Statistical analysis was performed using a Student’s *t*-test (one-tailed; *P<0.05, **P<0.01 and ***P<0.001; two-sample unequal variance). At least two independent measurements were performed for each experiment and the mean±s.d. of three biological replicates of a representative measurement is shown. All cells were grown and induced as described under experimental procedures.

**Figure 5:**
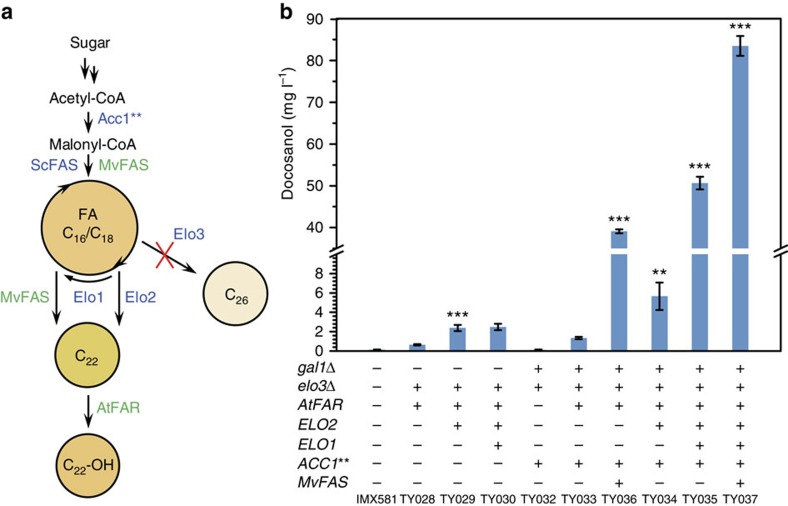
High levels of docosanol production by reconstructed efficient pathway. (**a**) Schematic overview describing the applied strategy for increasing precursor supply and targeted docosanol production. (**b**) Docosanol production in CEN.PK113-5D background with stepwise applied modifications. All strains were grown in minimal medium with 0.5% galactose. All genes were integrated into the genome except *mvfas*, which was expressed from the plasmid pSPGM2-MvFAS-AcpS. Statistical analysis was performed using a Student’s *t*-test (one-tailed; *P<0.05, **P<0.01 and ***P<0.001; two-sample unequal variance). At least two independent measurements were performed for each experiment and the mean±s.d. of three biological replicates of a representative measurement is shown.

These results clearly demonstrate that we were successful in establishing and combining three main platforms facilitating targeted VLCFAs and derived chemicals production in yeast (Fig. 1): (1) providing increased precursor supply for FA production, (2) tuning of yeast elongation system and choice of mycobacterial FAS I system for targeted chain length control of VLCFAs, and (3) choice of terminal enzyme and dynamic pathway control to generate the final product of interest. Our findings suggest that other chain length VLCFAs could be produced in a similar manner by tuning endogenous elongases, along with selection of the appropriate mycobacterial FAS I system. Depending on the background strains used and the terminal enzyme of choice, this technology would enable the production of various VLCFAs and derived products. These can be used for both bulk and specialty chemicals. In biobased production, the rate and yield on glucose are the main determinants for production costs, and hereby determining the cost competitiveness on the market. For low-priced bulk chemicals such as, for example, erucic acid, one has to operate near the theoretical yield, whereas for high-priced products, for example, waxes for cosmetics, lower yields are acceptable. At the same time, for products with higher chemical complexity more engineering has to be done to facilitate production. We show in this study that we were able to establish a production platform where we targeted several modifications, such as FA chain length and specific conversion to a C22 fatty alcohol, and even though we reach reasonable titres, further improvements are required to reach a commercial process.

However, our proof-of-principle demonstration that yeast can be used for production of long-chain fatty alcohols will facilitate the construction of microbial cell factories for the production of various new products in this area, in particular considering that industry favours the use of yeast as a robust and industrially established production host.

## Methods

### Strains and reagents

Primers, plasmids and *S. cerevisiae* strains used are listed in Supplementary Tables 4, 5 and 7. PrimeStar DNA polymerase was purchased from TaKaRa Bio. Taq DNA polymerase, restriction enzymes, DNA gel purification and plasmid extraction kits were purchased from Thermo Scientific. The Gibson Assembly Cloning Kit was purchased from NEB. Yeast plasmid Miniprep I kits were purchased from Zymo Research. All oligonucleotides (Supplementary Table 4) were synthesized at Sigma-Aldrich. All chemicals including analytical standards were purchased from Sigma-Aldrich, unless stated otherwise. All codon optimized heterologous genes were synthesized (Genscript) and listed in Supplementary Table 6.

### Strain cultivation

Yeast strains for preparation of competent cells were cultivated in YPD consisting of 10 g l^−1^ yeast extract (Merck Millipore), 20 g l^−1^ peptone (Difco) and 20 g l^−1^ glucose (Merck Millipore). Strains containing *URA3* marker-based plasmids or cassettes were selected on synthetic complete media without uracil, which consisted of 6.7 g l^−1^ yeast nitrogen base without amino acids (Formedium), 0.77 g l^−1^ CSM without uracil (Formedium), 20 g l^−1^ glucose (Merck Millipore) and 20 g l^−1^ agar (Merck Millipore). The *URA3* maker was removed and selected against on synthetic complete media+5-FOA plates, which contained 6.7 g l^−1^ yeast nitrogen base, 0.77 g l^−1^ CSM and 0.8 g l^−1^ 5-FOA. Strains containing the *kanMX* cassette were selected on YPD plates containing 200 mg l^−1^ G418 (Formedium). Shake flask batch fermentations for the production of fatty alcohols were carried out in minimal medium^10^,^45^ containing 30 g l^−1^ glucose with or without 0.5% galactose supplemented with 60 mg l^−1^ uracil if needed. Cultures were inoculated, from 24 h precultures, at initial OD_600_ of 0.05 in 15 ml minimal medium and cultivated at 200 r.p.m., 30 °C for 72 h. For Δ*fas1*/Δ*fas2* strain, C_16_ (5 mM palmitic acid) and C_18_ (5 mM stearic acid) free FAs were dissolved in ethanol/Tween 20 (1:1) to get 100 ml 100 × FA stock medium, then sterilized by membrane filtration were and added as described before^27^.

### Synthetic genes

*S. cerevisiae* codon-optimized synthetic genes of *cffar* (GenBank: AEO89345.1), *atfar* (NCBI reference sequence: NP_197642.1), *amfar* (NCBI accession number ADJ56408), *scfar* (NCBI accession number AF149917), *tafar* (TAA1a; NCBI accession number CAD30692), *acps* (NCBI reference sequence: WP_040539704.1) and *mvfas* (NCBI reference sequence: WP_003928293.1) were synthesized by GenScript, Inc. (Supplementary Table 6).

### Construction of *elo3*Δ∷ *kanMX*

Oligonucleotide primers are listed in Supplementary Table 4. To delete *ELO3*, the Del1 (LoxP-first half *kanMX* gene) fragment was amplified by primer pair pTY001/pTY002 using the plasmid pJET1.2-Del1 as template. The 502 bp upstream of the *ELO3* gene was amplified from CEN.PK113-11C genomic DNA with primer pair pTY005/pTY006. The upstream *ELO3* region was fused by fusion PCR with the Del1 fragment to obtain the fragment Upstream *ELO3*-LoxP-first half *kanMX*. The Del2 fragment (second half of kanMX gene (+overlap)-GAL-promoter-Cre.recombinase-LoxP) was amplified by primer pair pTY003/pTY004 using the plasmid pJET1.2-Del2 as a template. The 500 bp downstream of *ELO3* gene was amplified from CEN.PK113-11C genomic DNA with primer pair pTY007/pTY008. The downstream *ELO3* region was fused with the Del2 fragment to obtain Downstream *ELO3* second half of *kanMX* gene (+overlap)-GAL-promoter-Cre.recombinase-LoxP. Both fusion constructs were then used for co-transformation of strain JV03 to obtain strain TY001. The transformants were selected on G418 plates. Clones were verified by colony PCR using primer pairs pTY009/pTY002 and pTY010/pTY003. The *ELO2* gene was amplified from CEN.PK113-11C genomic DNA with primer pair pTY024/pTY025. The backbone vector pSPGM2 was digested by BamHI. The *ELO2* gene was ligated into the BamHI site of pSPGM2 to generate pELO2 by Gibson cloning. pELO2 was used to transform strain TY001 to generate TY002.

### Construction of expression plasmid pSPGM2-MvFAS-AcpS

The pSPGM2-MvFAS-AcpS was assembled on a yeast plasmid backbone pSPGM2 by using a modular pathway engineering strategy as described before^46^. In detail, the vector pSPGM2 was amplified by primer pair pTY011/pTY012. The whole expression cassette MvFAS-(GGGGS)_3_-AcpS was assembled using four parts: MvFAS2, MvFAS3, MvFAS4 and *acps*. These four parts have homologous overhangs among each other. In addition, MvFAS2 shared homologous overhangs with the *TEF1* promoter of pSPGM2. The *acps* part shared homologous overhangs with the *CYC1* terminator of pSPGM2. The MvFAS2 was amplified by primer pair pTY013/pTY014. The MvFAS3 was amplified by primer pair pTY015/pTY016. The MvFAS4 was amplified by primer pair pTY017/pTY018. The *acps* was amplified by primer pair pTY019/pTY020. The primer pTY019 has the 3*GGGGS sequence. All five parts were transformed into TDY7005 (Δ*fas1*/Δ*fas2*) and transformants were selected on YPD plates without any FA. Clones were verified by colony PCR. Subsequently, 3–4 clones with correct module integration were cultivated overnight in YPD liquid medium (Supplementary Fig. 6).

### Functional replacement of *ELO2/3* gene by MvFAS

The p415GPD-MvFAS-AcpS plasmid was assembled with a yeast plasmid backbone p415GPD by using a modular pathway engineering strategy as described before. In detail, the vector p415GPD was amplified by primers pTY022/pTY080. The whole expression cassette MvFAS-(GGGGS)_3_-AcpS was assembled in three parts: GPD-MvFAS2, MvFAS3 and MvFAS4-AcpS-CYC1t. These three parts have homologous regions between each other. The *GPD* (*TDH3*) promoter was amplified by primer pair pTY021/pTY022. The MvFAS2 was amplified by primer pair pTY023/pTY014. Then, the *GPD* promoter and MvFAS2 were fused together by overlapping PCR to obtain fragment GPD-MvFAS2. MvFAS3 was amplified with primer pair pTY015/pTY016. MvFAS4-AcpS-CYC1t was amplified by primer pair pTY017/pTY081 using pSPGM2-MvFAS-AcpS as template. Then, all the five parts were transformed into TDY7005 (Δ*elo2*/Δ*elo3*/pELO3) and transformants were selected on SD-LEU plates. These transformants were screened for growth on 5-FOA plates. Surviving clones were verified by colony PCR. Randomly, ten colonies were identified that had lost the plasmid that carried the *URA3* marker and the *ELO3* gene.

### Construction of FAR expression plasmids

Codon-optimized synthetic genes of *cffar*, *atfar*, *amfar*, *scfar* and *tafar* were amplified by primer pairs pTY026/pTY027, pTY028/pTY029, pTY034/pTY035, pTY030/pTY031 and pTY032/pTY033, respectively. These FAR fragments were each ligated into the SpeI/NotI site of pSPGM2 to generate pVLCFAlc03, pVLCFAlc02, pVLCFAlc01, pVLCFAlc04 and pVLCFAlc05 via Gibson cloning. These plasmids were employed to transform TY001 (JV03 Δ*elo3*) to generate the strains TY008, TY007, TY006, TY009 and TY010. The respective FAR fragments were also ligated into the SpeI/NotI site of pELO2 to generate pVLCFAlc08, pVLCFAlc07, pVLCFAlc06, pVLCFAlc09 and pVLCFAlc10 via Gibson cloning. These plasmids were used to transform TY001 (JV03 Δ*elo3*) to generate the strains TY013, TY012, TY011, TY014 and TY015 (Supplementary Fig. 7).

### Construction of plasmids for expression of *AtFAR*

The promoters *ADH2*p, *ICL1*p, *GAL1*p, *TEF1*p, *TDH3*p and *HXT7*p were amplified from CEN.PK113-11C genomic DNA with primer pairs pTY036/pTY038, pTY039/pTY041, pTY042/pTY044, pTY045/pTY060, pTY049/pTY062 and pTY047/pTY064 separately. The *atfar* gene was amplified by the primer pair pTY051/pTY052. The fragment *FBA1*t was amplified from CEN.PK113-11C genomic DNA with the primer pair pTY053/pTY054. The plasmid p416GPD was digested by SacI and KpnI, and the promoter *ADH2*p; the amplified *atfar* fragment and the *FBA1* terminator were ligated into the SacI/KpnI site of p416GPD to generate the plasmid pDynCon05 via Gibson assembly. The same strategy was used to construct plasmids pDynCon06, pDynCon03, pDynCon02, pDynCon01 and pDynCon04. These plasmids were used to transform TY001 (JV03 Δ*elo3*) to generate the strains TY020, TY021, TY018, TY017, TY016 and TY019. To construct another set of plasmids, the promoters *ADH2*p, *ICL1*p, *GAL1*p, *TEF1*p, *TDH3*p and *HXT7*p were amplified from CEN.PK113-11C genomic DNA with primer pairs pTY037/pTY038, pTY040/pTY041, pTY043/pTY044, pTY046/pTY060, pTY050/pTY062 and pTY048/pTY064. The *ELO3* gene was amplified from CEN.PK113-11C genomic DNA with primer pair pTY055/pTY056. The *HXT1*p promoter was amplified from CEN.PK113-11C genomic DNA with primer pair pTY057/pTY058. In the following, the fragments *ELO3*, *HXT1*p, *ADH2*p, *atfar* and *FBA1*t were ligated into the SacI /Hindlll site of the p416GPD vector to generate the plasmid pDynCon11. The same strategy was used to construct the plasmids pDynCon12, pDynCon09, pDynCon08, pDynCon07 and pDynCon10. These plasmids were used to transform TY001 (JV03 Δ*elo3*) to generate strains TY026, TY027, TY024, TY023, TY022 and TY025 (Supplementary Fig. 8).

### Reconstruction of the docosanol pathway

To select for specific genomic RNAs targeting for *GAL1* and *ELO3*, all potential gRNAs were compared with all potential off-targets in the entire CEN.PK113-7D genome using the Yeastriction webtool^34^, which is freely available at http://yeastriction.tnw.tudelft.nl. For deletion of *GAL1*, the 2 μm fragment of pROS13 was amplified by primer pair pTY067/pTY068. The linearized backbone of pROS13 was amplified by pTY102. The 2 μm fragment and the backbone of pROS13 and were fused using Gibson Assembly to generate the gRNA expression vector pROS13-gRNA:GAL1. To repair the double-strand break of *GAL1*, primers comprised of 2 × 60 bp sequences homologous to up- and downstream regions of *GAL1* were used. The correct colonies were confirmed by primer pair pTY042/pTY073. To repair the double-strand break of *GAL1* and integrate the ACC1** (*Acc1*^S659A,S1157A^) gene^38^, ACC1** was amplified by primer pair pTY071/pTY072, which comprised 100 bp overhang sequences homologous to up- and downstream regions of *GAL1*. The gRNA expression vector and repair fragment or primers were used to transform the IMX581 strain to generate strain TY031. Correct strains were confirmed by primer pairs pTY042/pTY075 and pTY073/pTY074. For deletion of *ELO3*, the 2 μm fragment of pROS10 was amplified by primer pair pTY069/pTY070. The linearized backbone of pROS10 was amplified by primer pTY102. The 2 μm fragment and the backbone of pROS13 were fused using Gibson Assembly to get the gRNA expression vector pROS10-gRNA:ELO3. The double-strand break of *ELO3* was repaired by primer pair pTY065/pTY066, which comprised 60 bp sequences homologous to both up- and downstream regions of *ELO3*. The gRNA expression vector and repair primers were employed to transform strain TY031 to obtain TY032. Correct colonies were confirmed by primer pair pTY076/pTY077. To repair the double-strand break of *ELO3* and integrate the *atfar* gene under control of the *GAL1* promoter, the upstream fragment of *ELO3* was amplified from CEN.PK113-11C genomic DNA with primer pair pTY094/pTY095. The *GAL1*p promoter was amplified from CEN.PK113-11C genomic DNA with primer pair pTY096/pTY097. The *atfar* gene was amplified by primer pair pTY087/pTY086 and the *FBA1*t terminator was amplified from CEN.PK113-11C genomic DNA with primer pair pTY088/pTY089. In addition, the downstream region of *ELO3* was amplified from CEN.PK113-11C genomic DNA with primer pair pTY091/pTY092. Then, the five fragments were fused together by overlapping fusion PCR to obtain integration cassette ELO3up-GAL1p-AtFAR-FBAt-ELO3dw. The gRNA expression vector and repair fragment were used to transform TY031 or IMX581 to generate the strains TY033 and TY028, respectively. To repair the double-strand break of *ELO3* and integrate the *atfar* gene and *ELO2* gene, the upstream fragment of *ELO3* was amplified from CEN.PK113-11C genomic DNA with primer pair pTY078/pTY079. In addition, the *CYC1* terminator was amplified from CEN.PK113-11C genomic DNA with primer pair pTY080/pTY081 and the *ELO2* gene was amplified from CEN.PK113-11C genomic DNA with primer pair pTY082/pTY083. The bidirectional promoter *GAL1*p/*GAL10*p was amplified from CEN.PK113-11C genomic DNA with primer pair pTY084/pTY085. *Atfar* was amplified by primer pair pTY086/pTY087 and the *FBA1*t terminator was amplified by primer pair pTY088/pTY089. The downstream region of *ELO3* was amplified from CEN.PK113-11C genomic DNA with primer pair pTY091/pTY092. Then, these fragments were fused together by overlapping PCR to generate the two integration cassettes ELO3up-CYC1t-ELO2-GAL10-GAL1p and GAL10-GAL1p-AtFAR-FBA1t-ELO3dw. The gRNA expression vector and repair fragment were used to transform TY031 or IMX581 to generate the strains TY034 and TY029, respectively. To repair the double-strand break of *ELO3* and integrate the *atfar* gene and *ELO2*, the upstream fragment of *ELO3* was amplified from CEN.PK113-11C genomic DNA with primer pair pTY073/pTY093. The *TDH2* terminator was amplified from CEN.PK113-11C genomic DNA with primer pair pTY103/pTY104. The *ELO1* gene was amplified from CEN.PK113-11C genomic DNA with primer pair pTY105/pTY106. The *GAL7* promoter was amplified from CEN.PK113-11C genomic DNA with primer pair pTY100/pTY101. The *CYC1* terminator was amplified from CEN.PK113-11C genomic DNA with primer pair pTY080/pTY081. The *ELO2* gene was amplified from CEN.PK113-11C genomic DNA with primer pair pTY082/pTY083. The bidirectional promoter *GAL1*p/*GAL1*p was amplified from CEN.PK113-11C genomic DNA with primer pair pTY084/pTY085. The *atfar* was amplified by primer pair pTY086/pTY087. The *FBA1*t terminator was amplified by primer pair pTY088/pTY089. The downstream region of *ELO3* was amplified from CEN.PK113-11C genomic DNA with primer pair pTY091/pTY092. Then, these fragments were fused together by overlapping PCR to obtain the two integration cassettes ELO3up-TDH2t-ELO1-GAL7p-CYC1t-ELO2 and ELO2-GAL10p-GAL1p-AtFAR-FBAt-ELO3dw. The gRNA expression vector and repair fragments were used to transform TY031 or IMX581 to generate strains TY035 and TY030, respectively. The strains TY033 and TY035 were transformed with plasmid pSPGM2-MvFAS-AcpS to generate the strains TY036 and TY037 (Supplementary Figs 9 and 10).

### Total FA identification and quantification using GC–MS

Briefly, cell pellets were collected from 20 ml cell culture and then freeze dried for 48 h. Ten milligrams or 40 OD_600_ of cell culture was prepared in an extraction tube. As extraction solvent, a solution of 1 ml hexane and 2 ml of 14% BF_3_ in MeOH was used, which contained heneicosylic acid (C21:0, FA) as an internal standard. Subsequently, samples were flushed with N_2_ gas (∼30 s) to remove air and the lid was closed tightly. The transesterification was performed using a microwave treatment by programming the heating programme to increase from room temperature to 120 °C (within 6 min) and then keep the temperature at 120 °C for 5 min. Two millilitres of Milli-Q (MQ) water were added after the samples had been cooled down to room temperature. Samples were vortexed (20 s) and centrifuged at 2,000 *g* for 5 min. The upper phase (hexane phase that contained the fatty acid methyl esters (FAMEs)) was transferred into a GC glass vials. The samples were analysed by gas chromatography (GC; Focus GC, Thermo Fisher Scientific, USA) equipped with a Zebron ZB-5MS GUARDIAN capillary column (30 m × 0.25 mm × 0.25 μm, Phenomenex) and a DSQII mass spectrometer (Thermo Fisher Scientific)^47^. The GC programme was as follows: initial temperature of 50 °C, hold for 5 min; ramp to 140 °C at a rate of 10 °C min^−1^ and hold for 10 min; ramp to 310 °C at a rate of 15 °C min^−1^ and hold for 7 min. The GC programme for fatty alcohol quantification was as follows: initial temperature of 45 °C hold for 2.5 min; ramp to 220 °C at a rate of 20 °C min^−1^ and hold for 2 min; ramp to 300 °C at a rate of 20 °C min^−1^ and hold for 5 min. The temperature of inlet, mass transfer line and ion source were kept at 250 °C, 300 °C and 230 °C, respectively. The flow rate of the carrier gas (helium) was set at 1.0 ml min^−1^ and data were acquired at full scan mode (50–650 *m*/*z*). Final quantification was performed with Xcalibur software.

### Fatty alcohol identification and quantification

For fatty alcohol identification and quantification, cell pellets were collected from 5 ml cell cultures and then freeze dried for 72 h. As extraction solvent, a 2:1 chloroform:methanol solution was used, which contained heneicosanol as internal fatty alcohol standard. The extracted fraction was dried by rotary evaporation and dissolved in ethyl acetate^10^. Quantification of fatty alcohols was performed on the same gas chromatography–mass spectrometry (GC–MS) system as used for FAME analysis or a GC–FID system (Thermo Fisher Scientific). The GC programme for fatty alcohol quantification was as follows: initial temperature of 45 °C hold for 2.5 min; then ramp to 220 °C at a rate of 20 °C min^−1^ and hold for 2 min; ramp to 300 °C at a rate of 20 °C min^−1^ and hold for 5 min. Especially for docosanol analysis, the following GC programme was used: initial temperature of 50 °C, hold for 5 min; then ramp to 140 °C at a rate of 10 °C min^−1^ and hold for 10 min; ramp to 310 °C at a rate of 15 °C min^−1^ and hold for 7 min.

### Data availability

The authors declare that all data supporting the findings of this study are available within the article and its Supplementary Information file or available from the corresponding author upon reasonable request.

## Additional information

**How to cite this article:** Yu, T. *et al*. Metabolic engineering of *Saccharomyces cerevisiae* for production of very long chain fatty acid-derived chemicals. *Nat. Commun.* **8,** 15587 doi: 10.1038/ncomms15587 (2017).

**Publisher’s note**: Springer Nature remains neutral with regard to jurisdictional claims in published maps and institutional affiliations.

## Supplementary Material

Supplementary Information Supplementary Figures, Supplementary Tables and Supplementary References
