# Author Correction: Metabolic engineering of *Saccharomyces cerevisiae* for production of very long chain fatty acid-derived chemicals

**DOI:** 10.1038/ncomms16220

**Published:** 2018-07-13

**Authors:** Tao Yu, Yongjin J. Zhou, Leonie Wenning, Quanli Liu, Anastasia Krivoruchko, Verena Siewers, Jens Nielsen, Florian David

Nature Communications 8: Article number: 15587; DOI: 10.1038/ncomms15587 (2017); Published: 05 26 2017, Updated: 07 13 2018

In the originally published version of this Article, financial support was not fully acknowledged. The PDF and HTML versions of the Article have now been corrected to include support from the European Union’s Horizon 2020 Framework Programme for Research and Innovation—Grant Agreement No. 720824.

