# Supplementary material for: Metabolic engineering of Saccharomyces cerevisiae for production of very long chain fatty acid-derived chemicals
Source: Nat Commun. 2017 May 26;8:15587. doi: 10.1038/ncomms15587 (PMC5458556; doi:10.1038/ncomms15587)
Supplement: Supplementary Information — Supplementary Figures, Supplementary Tables and Supplementary References [file ncomms15587-s1.pdf]

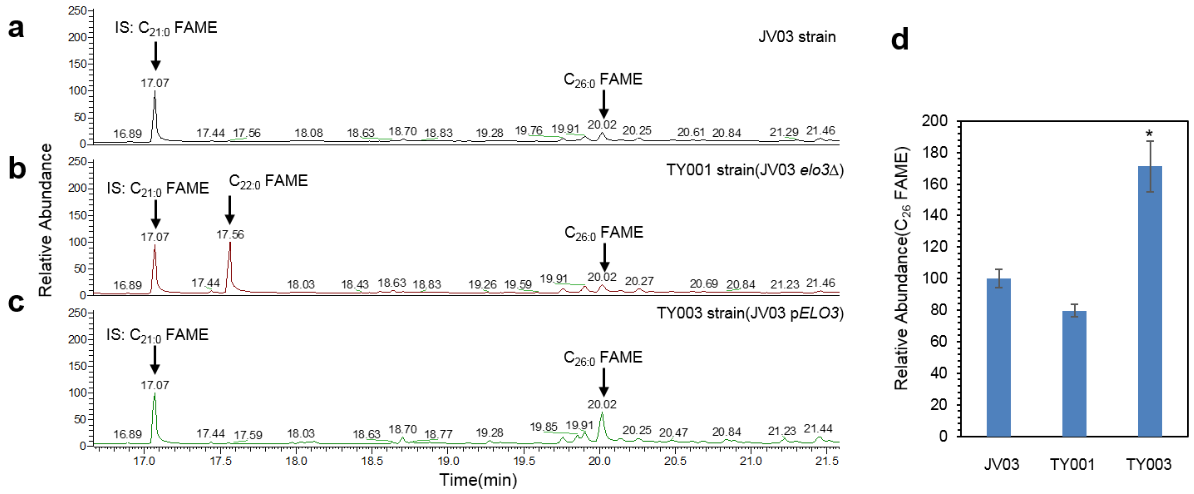

Supplementary Fig. 1. Fatty acid chain length profiles of VLCFA-producing JV03 and derived strains. Typical GC-MS total ion chromatograms (TIC) of total fatty acids extracted from JV03 cells **(a)**, TY001 (JV03 *elo3*Δ) **(b)** and strain TY003 (JV03 pELO3) **(c)**. **(d)** Relative quantification of total FA (C_{26} FAME) generated by yeast elongation system in strains JV03, TY001 and TY003. Statistical analysis was performed using a Student's t-Test (one-tailed; *, $p < 0.05$; **, $p < 0.01$ and ***, $p < 0.001$; Two-sample unequal variance). At least two independent measurements were performed for each experiment, and the the mean ± s.d of 3 biological replicates of a representative measurement is shown. Total fatty acids were identified by retention time and comparison with the mass spectral library. All cells were grown as described in experimental procedures.

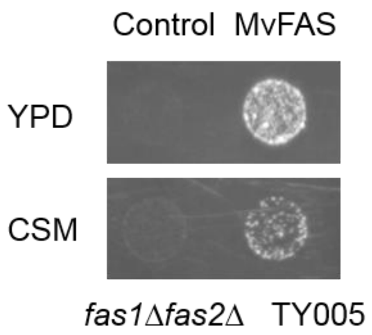

Supplementary Fig. 2. Complementation with the MvFAS restores the growth of a *fas1*Δ/*fas2*Δ strain. Spot assays of the *fas1*Δ/*fas2*Δ strain without or with expression of *mvfas* (strain TY005) on YPD or CSM plates. The plates were incubated at 30°C and recorded photographically 3 days after inoculation.

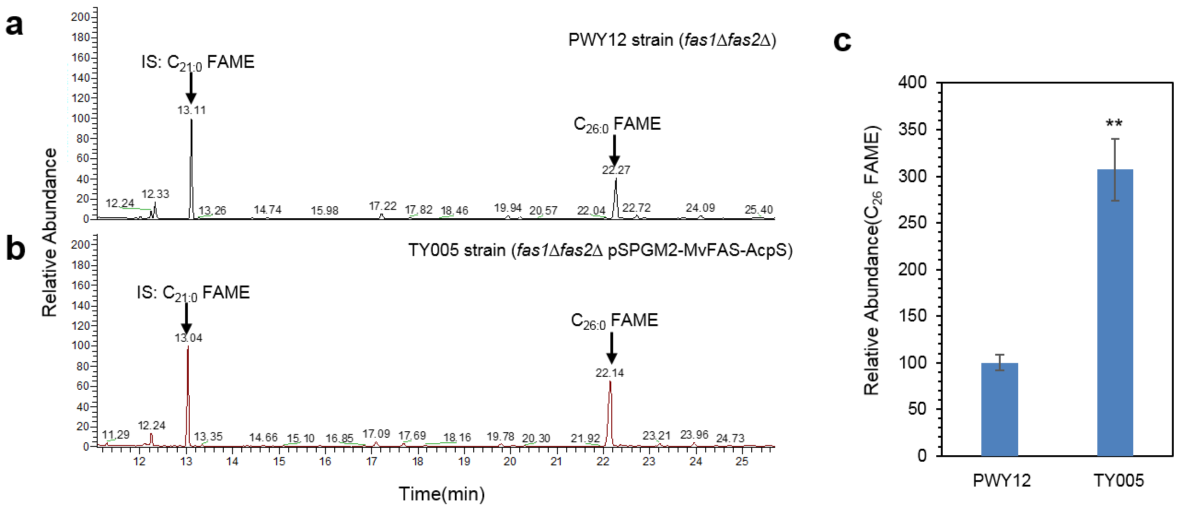

Supplementary Fig. 3. Fatty acid chain length profiles of VLCFA-producing *fas1*Δ/*fas2*Δ strains. Typical GC-MS total ion chromatograms (TIC) of total fatty acids extracted from PWY12 (*fas1*Δ/*fas2*Δ) **(a)**, or with plasmid pSPGM2-MvFAS-AcpS (strain TY005) **(b).** Total fatty acids were identified by retention time and comparison with the mass spectral library. **(c)** Relative quantification of total FA (C_{26} FAME) generated by strains PWY12 and TY005. Statistical analysis was performed using a Student's t-Test (one-tailed; *, $p < 0.05$; **, $p < 0.01$ and ***, $p < 0.001$; Two-sample unequal variance). At least two independent measurements were performed for each experiment, and the the mean ± s.d of 3 biological replicates of a representative measurement is shown. All cells were grown as described in experimental procedures.

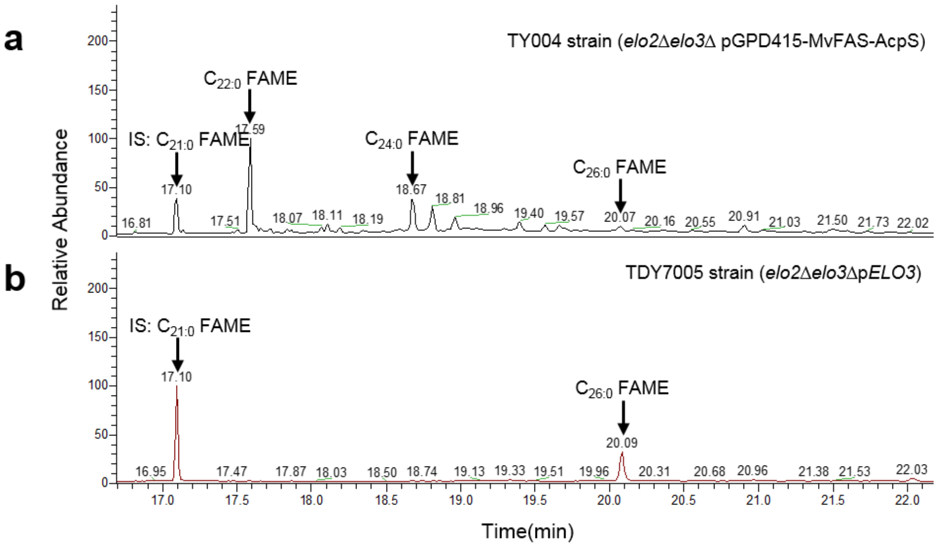

Supplementary Fig. 4. Fatty acid chain length profiles of VLCFA-producing *elo2*Δ/*elo3*Δ strains. Typical GC-MS total ion chromatograms (TIC) of total fatty acids extracted from TDY7005 (*elo2*Δ/*elo3*Δ) carrying p415GPD-MvFAS-AcpS (strain TY004) **(a)**, or the control plasmid pELO3 (strain TDY7005) **(b)**. Total fatty acids were identified by retention time and comparison with the mass spectral library. All cells were grown as described in experimental procedures.

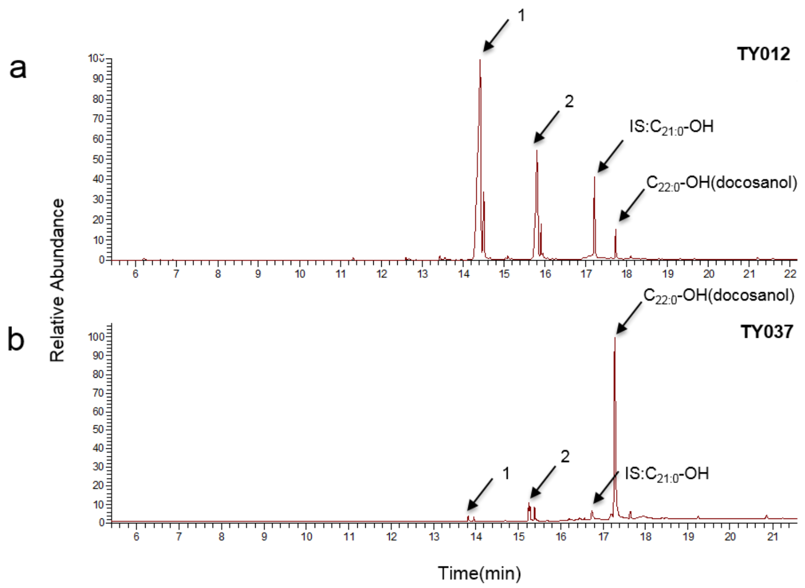

Supplementary Fig. 5. Fatty alcohol profiles of docosanol producing strains. **(a)** Typical GC-MS total ion chromatogram (TIC) of fatty alcohol extracted from starting strain TY012 (JV03 *elo3*Δ pELO2 pAtFAR). **(b)** Typical GC-FID total ion chromatograms (TIC) of fatty alcohol extracted from the best docosanol producer strain TY037 (MATa *ura3-52 can1Δ::cas9-natNT2 TRP1 LEU2 HIS3 gal1Δ*::*ACC1*** *elo3Δ::AtFAR ELO2 ELO1*/pSPGM2-MvFAS-AcpS) (Fig 5b). Both samples were spiked with the same amounts of internal standard. 1, $C_{16:0}$ and $C_{16:1}$ fatty acid. 2, $C_{18:0}$ and $C_{18:1}$ fatty acid. Fatty alcohols were identified by retention time and comparison with the mass spectral library. All cells were grown and induced as described in experimental procedures.

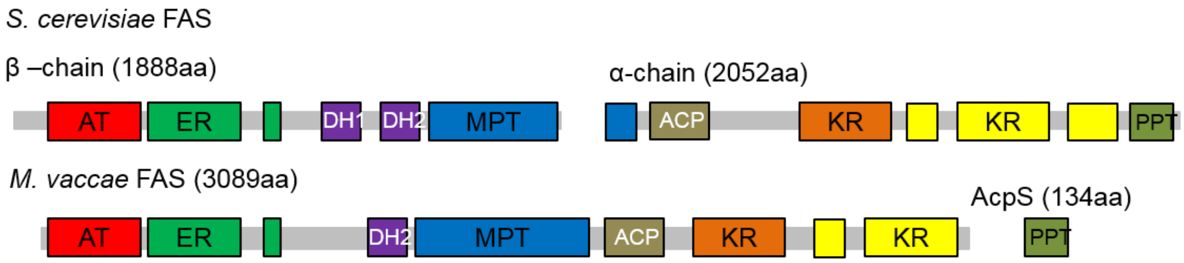

Supplementary Fig. 6. Schematic alignment of *MvFAS* and *ScFAS* drawn to scale (modified from[1]). The major domains of the two FASs are labelled. The PPT domain of *MvFAS* is encoded by gene *acps*. S. *cerevisiae* (*Saccharomyces cerevisiae*); *M. vaccae* (*Mycobacterium vaccae*); ACP (Acyl carrier protein); KR (Ketoacyl reductase); KS (Ketoacyl synthase); PPT (Phosphopantetheinyl transferase); AT (Acetyl transferase); ER (Enoyl reductase); DH (Dehydratase); MPT (Malonyl/palmitoyl transferase).

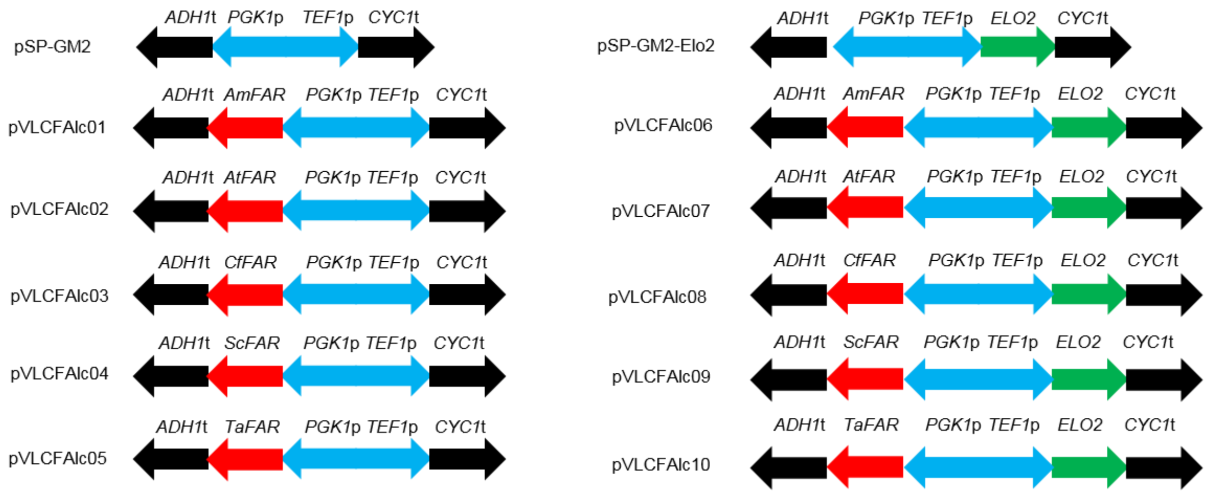

Supplementary Fig. 7. Genetic constructs used in FAR screening. Graphical representation of the *amfar, atfar, cffar, scfar* and *tafar* gene constructs used for screening C_{22} specifically FAR enzyme. The genetic sequences for *amfar, atfar, cffar, scfar* and *tafar* were codon optimized for expression in *S. cerevisiae* and synthesized by GenScript, Inc.

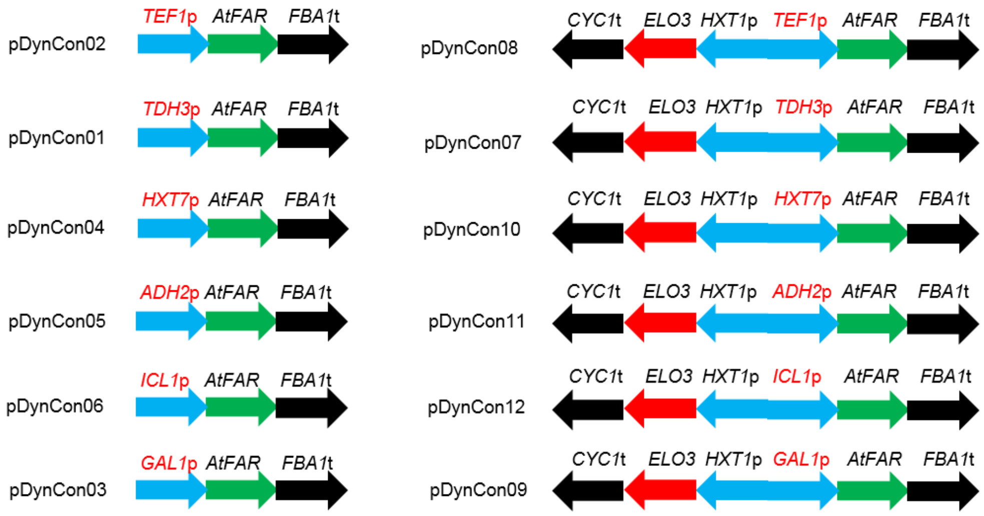

Supplementary Fig. 8. Genetic constructs used in promoter screening (dynamic control). Graphical representation of the *TEF1*p, *TDH3*p, *HXT7*p, *ADH2*p, *ICL1*p and *GAL1*p constructs used to screen for the best promoter combinations for the dynamic control system. All the promoters were amplified from *S. cerevisiae* CEN.PK113-5D genome DNA.

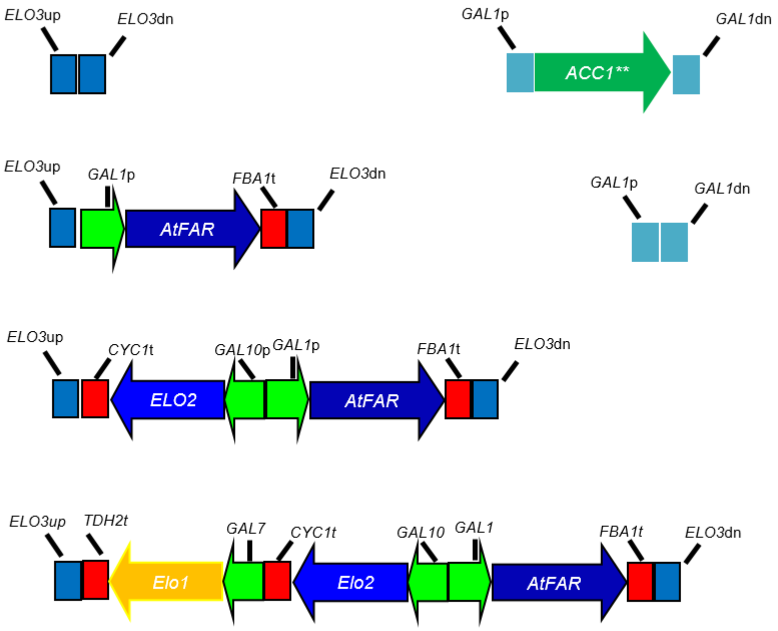

Supplementary Fig. 9. Schematic illustration summarizing the genetic arrangement of genomic integration constructs of target pathway genes. All the promoters and terminators were amplified from *S. cerevisiae* CEN.PK113-5D genome DNA.

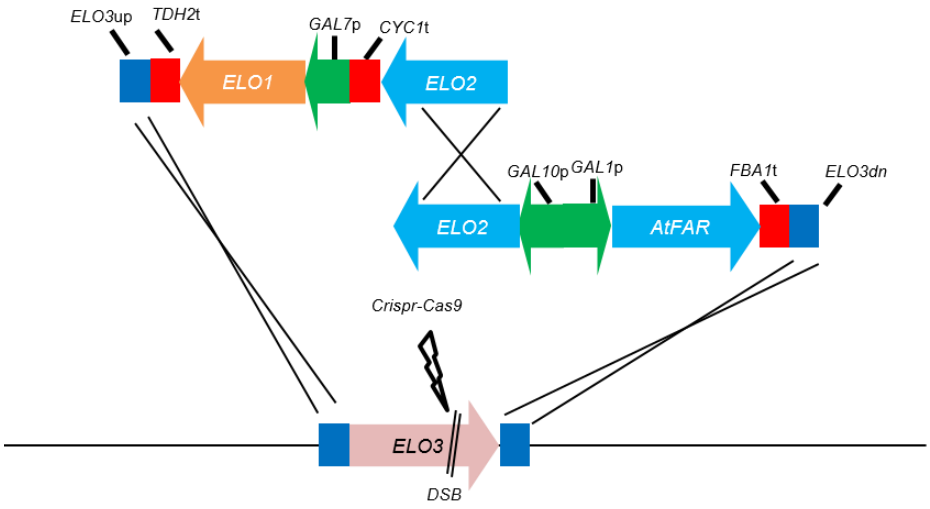

Supplementary Fig. 10. Schematic illustration of genome engineering strategy for gene deletion coupled with gene integration via CRISPR/Cas9.

Supplementary Table 1. Product specificity of FAS I system from different *Mycobacterium* species[2, 3]

species	Pyrolytic cleavage products of a-mycolate			species	Pyrolytic cleavage products of a-mycolate		
aurum	C22:0			thermoresistibile	C22:0	C24:0	
austroafricanum	C22:0			tokaiense	C22:0	C24:0	
brumane	C22:0			ulcerans	C22:0	C24:0	
diernhoferi	C22:0			vaccae	C22:0	C24:0	
fallax	C22:0			absessus		C24:0	
gilvum	C22:0			agri		C24:0	
neoaurum	C22:0			agri		C24:0	
parafortuitum	C22:0			asiaticum		C24:0	
alvei	C22:0	C24:0		avium subsp. Avium		C24:0	
chitae	C22:0	C24:0		avium subsp. Paratuberculosis		C24:0	
chubuense	C22:0	C24:0		chelonae		C24:0	
confluentis	C22:0	C24:0		farcinogenes		C24:0	
cookii	C22:0	C24:0		fortuitum		C24:0	
duvalii	C22:0	C24:0		kansasii		C24:0	
flavescens	C22:0	C24:0		komossense		C24:0	
gadium	C22:0	C24:0		lepramurim		C24:0	
gastri	C22:0	C24:0		marinum		C24:0	
gilvum	C22:0	C24:0		phlei		C24:0	
gordonae	C22:0	C24:0		shimoidei		C24:0	
haemophilum	C22:0	C24:0		simiae		C24:0	
hibernae	C22:0	C24:0		szulgai		C24:0	
intermedium	C22:0	C24:0		terrae		C24:0	
intracellulare	C22:0	C24:0		thermoresisibile lab		C24:0	
leprae	C22:0	C24:0		triviale		C24:0	
mucogenicum	C22:0	C24:0		celatum		C24:0	C26:0
nonchromogenicum	C22:0	C24:0		interjectum		C24:0	C26:0
obuense	C22:0	C24:0		malmoense		C24:0	C26:0
pulveris	C22:0	C24:0		microti		C24:0	C26:0
rhodesiae	C22:0	C24:0		tuberculosis		C24:0	C26:0
rhodesiae	C22:0	C24:0		africanum			C26:0
scrofulaceum	C22:0	C24:0		bovis			C26:0
smegmatis	C22:0	C24:0		bovis BCG			C26:0
sphagni	C22:0	C24:0		xenopi			C26:0

Supplementary Table 2. Fatty acyl-CoA reductase screening in different background strains

	JV03 *elo3Δ* strain						JV03 *elo3Δ* pELO2 strain				
FARs	*AmFAR*	*AtFAR*	*CfFAR*	*ScFAR*	*TaFAR*	control	*AmFAR*	*AtFAR*	*CfFAR*	*ScFAR*	*TaFAR*
OD_{600}	3.20	NS[*]	4.34	5.29	0.53	5.41	3.15	0.29	3.13	3.47	3.46
Docosanol(mg/L)	ND[ǂ]	ND	ND	ND	ND	ND	ND	1.13	ND	ND	ND
Strain name	TY006	TY007	TY008	TY009	TY010	TY001	TY011	TY012	TY013	TY014	TY015

ND[*]: Not detected; NS[ǂ]: No survival colony

Supplementary Table 3. Evaluation of different designs for dynamic control strategies

	JV03 *elo3Δ*							JV03 *elo3Δ HXT1*p-*ELO3*					
Promoters	*TDH3*p	*TEF1*p	*GAL1*p	*HXT7*p	*ADH2*p	*ICL1*p	control	*TDH3*p	*TEF1*p	*GAL1*p	*HXT7*p	*ADH2*p	*ICL1*p
OD_{600}	0	0	6.59±0.19	0	0	0	8.71±0.38	7.86±0.20	7.7±0.28	8.45±0.25	8.11±0.47	7.21±1.4	8.43±0.12
Docosanol(mg/L)	0	0	4.19±0.20	0	0	0	0	0	0	0	0.80±0.07	0.15±0.02	0
Docosanol(mg/OD_{600})	0	0	12.85±0.55	0	0	0	0	0	0	0	2.09±0.19	0.54±0.04	0
Strains	TY016	TY017	TY018	TY019	TY020	TY021	TY001	TY022	TY023	TY024	TY025	TY026	TY027

Supplementary Table 4. Primers used in this study

Primer	Description	Sequence(5´-3´)
pTY001	Del1-F	GACAACCCTTAATTACCGTTCGTATAATG
pTY002	Del1-R	CAACCTATTAATTTCCCCTCGTCA
pTY003	Del2-F	GGCAAAACAGCATTCCAGGT
pTY004	Del2-R	GGATCTGATATACCGTTCGTATAGCATACATTATACGAAGTTATTCACTATAGGGCGAATTGGGTAC
pTY005	Elo3up-F	AGCAGAATTATGAATAATAAACATTGATGACAATAG
pTY006	Elo3up-R	ACATTATACGAACGGTAATTAAGGGTTGTCGACTGTTTATGTTTCGTAAACAAACGG
pTY007	Elo3dw-F Primer	TGTATGCTATACGAACGGTATATCAGATCC ATA GGA AGC GAG AAT TTT TGA CAG CG
pTY008	Elo3dw-R Primer	TAGATAATGATGCTGGGCCGATAATTAG
pTY009	Chromosome XII before Elo3JD-F	TGCGTCGTTAGATTTCTGGGGTTAAGATAGCAAC
pTY010	Chromosome XII behind Elo3JD-R	TATTGGAGAGCTAGAACAACCAATGAACTTCATTGAAGAG
pTY011	p-vectorBamH1-R	TGTCATTGTTTTGTGAGTCGTATTACGGATCC
pTY012	p-vectorSma1fusion-F	TTGCAGTATTGGAAGAAAGATAAGCGTCGACATGGAACAGAAG
pTY013	P-MvFAS2-Gibson-F	GGATCCGTAATACGACTCACAAAACAATGACAATCAACGATCAACATAG
pTY014	P-MvFAS2-Gibson-R	TGTATGATCAGCGACCTTTTCTGGGTCCCATTCAACGGAGA
pTY015	P-MvFAS3-Gibson-F	TCTCCGTTGAATGGGACCCAGAAAAGGTCGCTGATCATACA
pTY016	P-MvFAS3-Gibson-R	GCAATACTACCTTTGGAGGCACCTGTGACTACTGCAACTTCATCA
pTY017	P-MvFAS4-Gibson-F	TGATGAAGTTGCAGTAGTCACAGGTGCCTCCAAAGGTAGTATTGC
pTY018	P-MvFAS4-AcpS-R	CTGAACCGCCACCACCTGATCTTCTGTAAACACCATTTTCAC
pTY019	AcpSfusion-F	TCAGGTGGTGGCGGTTCAGGCGGAGGTGGCTCTGGCGGTGGCGGATCGATGTTGCCAGTCGGTATAGTT
pTY020	AcpSfusion-R	CTTCTGTTCCATGTCGACGCTTATCTTTCTTCCAATACTGCAA
pTY021	GPD-F	ATCATTATCAATACTGCCATTTC
pTY022	GPD-R	CTATGTTGATCGTTGATTGTCATTAGTTCTAGATCCGTCGAAAC
pTY023	MvFAS-fragment2-F	GTTTCGACGGATCTAGAACTAATGACAATCAACGATCAACATAG
pTY024	ELO2-F	CATAGCAATCTAATCTAAGTTTTAATTACAAGGATCAAAACAATGAATTCACTCGTTACTCAATA
pTY025	ELO2-R	TCGACGCCCGGGCCCTATAGTGAGTCGTATTACGGATCTTACCTTTTTCTTCTGTGTTGAG
pTY026	CfFAR-F	ATCTTATCGTCGTCATCCTTGTAATCCATCGATACTAGTTTACATGTATCTTCTTGAACCTG
pTY027	CfFAR-R	TCTACTTTTTACAACAAATATAAAACAAGCGGCCGCAAAACAATGGAAGTTAACAACAACTCAC
pTY028	AT5g22500-F	ATCTTATCGTCGTCATCCTTGTAATCCATCGATACTAGTTTATTGTTTCAAAACATGTGTGAT
pTY029	AT5g22500-R	TCTACTTTTTACAACAAATATAAAACAAGCGGCCGCAAAACAATGGAATCTAATTGTGTTCAATTC
pTY030	ScFAR-F	ATCTTATCGTCGTCATCCTTGTAATCCATCGATACTAGTGTTCAATACATGTTCAACGACA

pTY031	ScFAR-R	TCTACTTTTTACAACAAATATAAAACAAGCGGCCGCAAAACAATGGAAGAAATGGGTTCAATCT
pTY032	TaFAR-F	ATCTTATCGTCGTCATCCTTGTAATCCATCGATACTAGTTTTCAACATGTATTTTATGACACC
pTY033	TaFAR-R	TCTACTTTTTACAACAAATATAAAACAAGCGGCCGCAAAACAATGGTTGATACATTGTCTGAAG
pTY034	AmFAR-F	ATCTTATCGTCGTCATCCTTGTAATCCATCGATACTAGTAATTTGATAGTAAGACGTGTAAG
pTY035	AmFAR-R	TCTACTTTTTACAACAAATATAAAACAAGCGGCCGCAAAACAATGTCAACTATTTCCGATAATCAATG
pTY036	Kpn1ADH2p-F	CAGTGAGCGCGCGTAATACGACTCACTATAGGGCGAATTGGCAAAACGTAGGGGCAAACA
pTY037	HXT1ADH2p-F	TAGTCGGTCGATATGAATTGAAGTTTCATTGTGGCGCAAAACGTAGGGGCAAACA
pTY038	ADH2p-R	GTCTTATTACCCAAGAATTGAACACAATTAGATTCCATTGTTTTTGTGTATTACGATATAGTTAATAG
pTY039	Kpn1ICL1p-F	CAGTGAGCGCGCGTAATACGACTCACTATAGGGCGAATTGTGGAAATGTAAAGGATAATGAGTG
pTY040	HXT1ICL1p-F	TAGTCGGTCGATATGAATTGAAGTTTCATTGTGGCTGGAAATGTAAAGGATAATGAGTG
pTY041	ICL1p-R	GTCTTATTACCCAAGAATTGAACACAATTAGATTCCATTGTTTTCGTTGACTTTTTGTTATGTTATG
pTY042	Kpn1GAL1p-F	CAGTGAGCGCGCGTAATACGACTCACTATAGGGCGAATTGCGGATTAGAAGCCGCCGAG
pTY043	HXT1GAL1p-F	TAGTCGGTCGATATGAATTGAAGTTTCATTGTGGCCGGATTAGAAGCCGCCGAG
pTY044	GAL1p-R	GTCTTATTACCCAAGAATTGAACACAATTAGATTCCATTGTTTTCTCCTTGACGTTAAAGTATAGAG
pTY045	Kpn1TEF1p-F	CAGTGAGCGCGCGTAATACGACTCACTATAGGGCGAATTGATAGCTTCAAAATGTTTCTACTC
pTY046	HXT1TEF1p-F	TAGTCGGTCGATATGAATTGAAGTTTCATTGTGGCATAGCTTCAAAATGTTTCTACTC
pTY047	Kpn1HXT7-F	CAGTGAGCGCGCGTAATACGACTCACTATAGGGCGAATTGAATAGTACTCTCATCGCTAAGATC
pTY048	HXT1HXT7-F	TAGTCGGTCGATATGAATTGAAGTTTCATTGTGGCATAGCTTCAAAATGTTTCTACTC
pTY049	Kpn1TDH3p-F	CAGTGAGCGCGCGTAATACGACTCACTATAGGGCGAATTGTCGAGTTTATCATTATCAATACTG
pTY050	HXT1TDH3p-F	TAGTCGGTCGATATGAATTGAAGTTTCATTGTGGCATAGCTTCAAAATGTTTCTACTC
pTY051	AtFAR-F	ATGGAATCTAATTGTGTTCAATTC
pTY052	AtFARFBA1-R	AAACTATATCAATTAATTTGAATTAACTTATTGTTTCAAAACATGTGTGATC
pTY053	5FBA1-F	GATCACACATGTTTTGAAACAATAAGTTAATTCAAATTAATTGATATAGTTT

pTY054	5FBA1-R	CAAGCGCGCAATTAACCCTCACTAAAGGGAACAAAAGCTGAGTAAGCTACTATGAAAGACTT
pTY055	Hind111Elo3-F	GACATAACTAATTACATGACTCGAGGTCGACGGTATCGATTTAAGCTTTCCTGGAAGAGAC
pTY056	Hind111Elo3-R	CAACTAGTTGATATACGTAAAATCAAAACAATGAACACTACCACATCTACTG
pTY057	3HXT1p-F	CAGTAGATGTGGTAGTGTTCATTGTTTTGATTTTACGTATATCAACTAGTTG
pTY058	3HXT1p-R	GCCACAATGAAACTTCAATTCA
pTY059	HXT1TEF1p-F	TAGTCGGTCGATATGAATTGAAGTTTCATTGTGGCATAGCTTCAAAATGTTTCTACTC
pTY060	TEF1p-R	GTCTTATTACCCAAGAATTGAACACAATTAGATTCCATTTTGTAATTAAAACTTAGATTAG
pTY061	HXT1TDH3p-F	TAGTCGGTCGATATGAATTGAAGTTTCATTGTGGC TCGAGTTTATCATTATCAATACTG
pTY062	TDH3-R	GTCTTATTACCCAAGAATTGAACACAATTAGATTCCATTTTGTTTGTTTATGTGTGTTTATTC
pTY063	HXT1HXT7-F	TAGTCGGTCGATATGAATTGAAGTTTCATTGTGGCAATAGTACTCTCATCGCTAAGATC
pTY064	HXT7-R	GTCTTATTACCCAAGAATTGAACACAATTAGATTCCATTTTTTGATTAAAATTAAAAAAACT
pTY065	Elo3repair-F	AGCTTACTTCTAGTTTATTTATTCGGCTTTTTTCCGTTTGTTTACGAAACATAAACAGTCATAGGAAGCGAGAATTTTTGACAGCGAATGAAAAAGAAAAAAATTAAATGTTAACATTTT
pTY066	Elo3repair-R	AAAATGTTAACATTTAATTTTTTTCTTTTTCATTCGCTGTCAAAAATTCTCGCTTCCTATGACTGTTTATGTTTCGTAAACAAACGGAAAAAAGCCGAATAAATAAACTAGAAGTAAGCT
pTY067	GAL1-gRNA1	TGCGCATGTTTCGGCGTTCGAAACTTCTCCGCAGTGAAAGATAAATGATCAACCAACTATAATTTAAGAGGTTTTAGAGCTAGAAATAGCAAGTTAAAATAAGGCTAGTCCGTTATCAAC
pTY068	GAL1-gRNA2	TGCGCATGTTTCGGCGTTCGAAACTTCTCCGCAGTGAAAGATAAATGATCACTAAAGCCCTGTTTCTTATGTTTTAGAGCTAGAAATAGCAAGTTAAAATAAGGCTAGTCCGTTATCAAC
pTY069	ELO3-gRNA1	TGCGCATGTTTCGGCGTTCGAAACTTCTCCGCAGTGAAAGATAAATGATCTACATCCAATCTTACAAGAAGTTTTAGAGCTAGAAATAGCAAGTTAAAATAAGGCTAGTCCGTTATCAAC
pTY070	ELO3-gRNA2	TGCGCATGTTTCGGCGTTCGAAACTTCTCCGCAGTGAAAGATAAATGATCGTAGTTATCCTACTAAACTTGTTTTAGAGCTAGAAATAGCAAGTTAAAATAAGGCTAGTCCGTTATCAAC
pTY071	ACC1repair-F	CTACTCGTTATTATTGCGTATTTTGTGATGCTAAAGTTATGAGTAGAAAAAAATGAGAAGTTGTTCTGAACAAAGTAAAAAAAAGAAGTATACTTATTTCAAAGTCTTCAACAATTTTTC
pTY072	ACC1repair-R	TTTCGGTTTGTATTACTTCTTATTCAAATGTCATAAAAGTATCAACAAAAAATTGTTAATATACCTCTATACTTTAACGTCAAGGAGAAAAAACTATAATGAGCGAAGAAAGCTTATTCG
pTY073	ACC1GAL1-upJDF	ACAAAGGGTTCTCGTAGAGTC

pTY074	ACC1GAL1-upJDR	AGGAACTGGAATGGACCGAG
pTY075	ACC1-DWjdF	CTAGTTTATCTACTGTATTGAGG
pTY076	Elo3 JDF	GTACTGCTACGAGGATTATCG
pTY077	Elo3 JDR	GCAATTTCATTAGAGATAGTGG
pTY078	ELO3up-F	CTGGGGTTAAGATAGCAACG
pTY079	ELO3up-R	CGCTCGAAGGCTTTAATTTGCGGCCGGTACCCGACTGTTTATGTTTCGTAAACAAAC
pTY080	CYC1-F	GATACCGTCGACCTCGAGTC
pTY081	CYC1-R	GGGTACCGGCCGCAAATTAAAG
pTY082	Elo2-F	CTAATTACATGACTCGAGGTCGACGGTATC TTACCTTTTTCTTCTGTGTTG
pTY083	Elo2-R	CAAAAAAAAAGTAAGAATTTTTGAAAATTCAATATAAAAAACAATGAATTCACTCGTTACTC
pTY084	Gal1andGal10-F	TTATATTGAATTTTCAAAAATT
pTY085	Gal1andGal10-R	TATAGTTTTTTCTCCTTGACGT
pTY086	AtFAR-F	CTATACTTTAACGTCAAGGAGAAAAAACTATAAAAACAATGGAATCTAATTGTGTTCAATTC
pTY087	AtFAR-R	CTCATTAAAAAACTATATCAATTAATTTGAATTAACTTATTGTTTCAAAACATGTGTGATC
pTY088	FBA1-F	GTTAATTCAAATTAATTGATATAG
pTY089	FBA1-R	AGTAAGCTACTATGAAAGACTTTAC
pTY090	Elo3updown-F	CGGCTTTTTTCCGTTTGTTTACGAAACATAAACAGTC ATAGGAAGCGAGAATTTTTGAC
pTY091	Elo3down-F	CTTCGAGTTCTTTGTAAAGTCTTTCATAGTAGCTTACTATAGGAAGCGAGAATTTTTGAC
pTY092	Elo3down-R	AGTTACTTGATATCCATGTTCC
pTY093	Elo3up-TDH2-R	CTTAGTATCACACTAATTGGCTTTTCGCGACTGTTTATGTTTCGTAAACAAAC
pTY094	Elo3up-Fxin	CTGGGGTTAAGATAGCAACG
pTY095	Elo3up-Rxin	GACTGTTTATGTTTCGTAAACAAAC
pTY096	Elo3upGAL1p-F	GCTTTTTTCCGTTTGTTTACGAAACATAAACAGTCCGGATTAGAAGCCGCCGAG
pTY097	GAL1p-R	TATAGTTTTTTCTCCTTGACGTT
pTY098	GAL7GAL1p-F	GTGCATATTTTCAAGAAGGATAGTAAGCTGGCAAACGGATTAGAAGCCGCCGAG
pTY099	ELO3upcyc-R	CGCTCGAAGGCTTTAATTTGCGGCCGGTACCCGACTGTTTATGTTTCGTAAACAAAC
pTY100	GAL7-F	TTTTGAGGGAATATTCAACTGTT
pTY101	GAL7 CYC1-R	CGCTCGAAGGCTTTAATTTGCGGCCGGTACCCTTTGCCAGCTTACTATCCTTC
pTY102	primer 6005	GATCATTTATCTTTCACTGCGGAGAAG
pTY103	TDH2t-F	ATTTAACTCCTTAAGTTACTTTAATGATTTAG
pTY104	TDH2t-R	GCGAAAAGCCAATTAGTGTGATAC
pTY105	Elo1-F	CTAAATCATTAAAGTAACTTAAGGAGTTAAATTTAATTGTTTTTGTTGATCTTCTTC

pTY106	Elo1-R	GATAAAAAAAAACAGTTGAATATTCCCTCAAAAAAAACAATGGTAAGTGATTGGAAAAATTTTTG

Supplementary Table 5 *S. cerevisiae* strains used in this study.

Strain	Genotype and plasmid	Source
JV03	MATa MAL2-8c *SUC2 ura3-52 HIS3 are1Δ dga1Δ are2Δ lro1Δ pox1Δ*	Ref[4]
TY001	MATa MAL2-8c *SUC2 ura3-52 HIS3 are1Δ dga1Δ are2Δ lro1Δ pox1Δ elo3Δ*	This work
TY002	MATa MAL2-8c *SUC2 ura3-52 HIS3 are1Δ dga1Δ are2Δ lro1Δ pox1Δ elo3Δ*/pELO2	This work
TY003	MATa MAL2-8c *SUC2 ura3-52 HIS3 are1Δ dga1Δ are2Δ lro1Δ pox1Δ*/pELO3	This work
TDY7005	Mata *lys2 ura3–52 trp1Δ leu2Δ elo2Δ::KAN elo3Δ::TRP1*/pELO3	Ref[5]
TY004	Mata *lys2 ura3–52 trp1Δ leu2Δ elo2Δ::KAN elo3Δ::TRP1*/pGPD415-MvFAS-AcpS	This work
PWY12	MATα *ura3Δ leu2Δ his3Δ trp1Δ can1Δ fas1Δ::HIS3 fas2Δ::LEU2*	Ref[6]
TY005	MATα *ura3Δ leu2Δ his3Δ trp1Δ can1Δ fas1Δ::HIS3 fas2Δ::LEU2*/pSPGM2-MvFAS-AcpS	This work
TY006	MATa MAL2-8c *SUC2 ura3-52 HIS3 are1Δ dga1Δ are2Δ lro1Δ pox1Δ elo3Δ*/pVLCFAlc01	This work
TY007	MATa MAL2-8c *SUC2 ura3-52 HIS3 are1Δ dga1Δ are2Δ lro1Δ pox1Δ elo3Δ*/pVLCFAlc02	This work
TY008	MATa MAL2-8c *SUC2 ura3-52 HIS3 are1Δ dga1Δ are2Δ lro1Δ pox1Δ elo3Δ*/pVLCFAlc03	This work
TY009	MATa MAL2-8c *SUC2 ura3-52 HIS3 are1Δ dga1Δ are2Δ lro1Δ pox1Δ elo3Δ*/pVLCFAlc04	This work
TY010	MATa MAL2-8c *SUC2 ura3-52 HIS3 are1Δ dga1Δ are2Δ lro1Δ pox1Δ elo3Δ*/pVLCFAlc05	This work
TY011	MATa MAL2-8c *SUC2 ura3-52 HIS3 are1Δ dga1Δ are2Δ lro1Δ pox1Δ elo3Δ*/pVLCFAlc06	This work
TY012	MATa MAL2-8c *SUC2 ura3-52 HIS3 are1Δ dga1Δ are2Δ lro1Δ pox1Δ elo3Δ*/pVLCFAlc07	This work
TY013	MATa MAL2-8c *SUC2 ura3-52 HIS3 are1Δ dga1Δ are2Δ lro1Δ pox1Δ elo3Δ*/pVLCFAlc08	This work
TY014	MATa MAL2-8c *SUC2 ura3-52 HIS3 are1Δ dga1Δ are2Δ lro1Δ pox1Δ elo3Δ*/pVLCFAlc09	This work
TY015	MATa MAL2-8c *SUC2 ura3-52 HIS3 are1Δ dga1Δ are2Δ lro1Δ pox1Δ elo3Δ*/pVLCFAlc10	This work
TY016	MATa MAL2-8c *SUC2 ura3-52 HIS3 are1Δ dga1Δ are2Δ lro1Δ pox1Δ elo3Δ*/pDynCon01	This work
TY017	MATa MAL2-8c *SUC2 ura3-52 HIS3 are1Δ dga1Δ are2Δ lro1Δ pox1Δ elo3Δ*/pDynCon02	This work
TY018	MATa MAL2-8c *SUC2 ura3-52 HIS3 are1Δ dga1Δ are2Δ lro1Δ pox1Δ elo3Δ*/pDynCon03	This work
TY019	MATa MAL2-8c *SUC2 ura3-52 HIS3 are1Δ dga1Δ are2Δ lro1Δ pox1Δ elo3Δ*/pDynCon04	This work
TY020	MATa MAL2-8c *SUC2 ura3-52 HIS3 are1Δ dga1Δ are2Δ lro1Δ pox1Δ elo3Δ*/pDynCon05	This work
TY021	MATa MAL2-8c *SUC2 ura3-52 HIS3 are1Δ dga1Δ are2Δ lro1Δ pox1Δ elo3Δ*/pDynCon06	This work

TY022	MATa MAL2-8c *SUC2 ura3-52 HIS3 are1Δ dga1Δ are2Δ lro1Δ pox1Δ elo3Δ*/pDynCon07	This work
TY023	MATa MAL2-8c *SUC2 ura3-52 HIS3 are1Δ dga1Δ are2Δ lro1Δ pox1Δ elo3Δ*/pDynCon08	This work
TY024	MATa MAL2-8c *SUC2 ura3-52 HIS3 are1Δ dga1Δ are2Δ lro1Δ pox1Δ elo3Δ*/pDynCon09	This work
TY025	MATa MAL2-8c *SUC2 ura3-52 HIS3 are1Δ dga1Δ are2Δ lro1Δ pox1Δ elo3Δ*/pDynCon10	This work
TY026	MATa MAL2-8c *SUC2 ura3-52 HIS3 are1Δ dga1Δ are2Δ lro1Δ pox1Δ elo3Δ*/pDynCon11	This work
TY027	MATa MAL2-8c *SUC2 ura3-52 HIS3 are1Δ dga1Δ are2Δ lro1Δ pox1Δ elo3Δ*/pDynCon12	This work
IMX581	MATa *ura3-52 can1Δ::cas9-natNT2 TRP1 LEU2 HIS3*	Ref[7]
TY028	MATa *ura3-52 can1Δ::cas9-natNT2 TRP1 LEU2 HIS3 elo3Δ::AtFAR*/pROS10-gRNA:elo3	This work
TY029	MATa *ura3-52 can1Δ::cas9-natNT2 TRP1 LEU2 HIS3 elo3Δ::AtFAR ELO2*/pROS10-gRNA:elo3	This work
TY030	MATa *ura3-52 can1Δ::cas9-natNT2 TRP1 LEU2 HIS3 elo3Δ::AtFAR ELO2 ELO1*/pROS10-gRNA:elo3	This work
TY031	MATa *ura3-52 can1Δ::cas9-natNT2 TRP1 LEU2 HIS3 gal1Δ::ACC1**/*pROS13-gRNA:gal1	This work
TY032	MATa *ura3-52 can1Δ::cas9-natNT2 TRP1 LEU2 HIS3 gal1Δ::ACC1** elo3Δ*/pROS10-gRNA:elo3	This work
TY033	MATa *ura3-52 can1Δ::cas9-natNT2 TRP1 LEU2 HIS3 gal1Δ::ACC1** elo3Δ::AtFAR*/pROS10-gRNA:elo3	This work
TY034	MATa *ura3-52 can1Δ::cas9-natNT2 TRP1 LEU2 HIS3 gal1Δ::ACC1** elo3Δ::AtFAR ELO2*/pROS10-gRNA:elo3	This work
TY035	MATa *ura3-52 can1Δ::cas9-natNT2 TRP1 LEU2 HIS3 gal1Δ::ACC1** elo3Δ::AtFAR ELO2 ELO1*/pROS10-gRNA:elo3	This work
TY036	MATa *ura3-52 can1Δ::cas9-natNT2 TRP1 LEU2 HIS3 gal1Δ::ACC1** elo3Δ::AtFAR*/pSPGM2-MvFAS-AcpS	This work
TY037	MATa *ura3-52 can1Δ::cas9-natNT2 TRP1 LEU2 HIS3 gal1Δ::ACC1** elo3Δ::AtFAR ELO2 ELO1*/pSPGM2-MvFAS-AcpS	This work

Note: For PWY12, in detail, for the Δ*fas1*::*HIS3* disruption, about 1.2 kb of the *FAS1* upstream region together with 1.4 kb of the N-terminal part of the *FAS1* reading frame were replaced by the *HIS3* insertion marker. Similarly, 0.9kb of the *FAS2* upstream region together with 1.3 kb of the N-terminal part of the *FAS2* reading frame were replaced by the *LEU2* insertion giving the *Δfas2::LEU2* construct.

Supplementary Table 6. Codon optimized genes used in this study.

Synthesized genes	Sequence (5’-3’)
MvFAS	ATGACAATCAACGATCAACATAGAGCCGCCGCTGGTAGTGACTCAAGAACTGGTCAAGACTT<br>CGAACCTTTATCTGGTACACACGCCTTGGTAGATAGATTACATGCTGGTGAACCATACGCTGT<br>CGCATTTGGTGGTCAAGGTGGTCCTTGGTTAGAAAATTTGGAAGAATTAGTTAACTCAGCTGG<br>TATCGAATCTGAAATTTCACAATTGGTAGCCGAAGCTGAATTAATGTTGGAACCATTGGCTAG<br>AGAATTAGTTGTAGTCAGACCAATTGGTTTCGAACCTATGAAGTGGATAAGAGCATTAGCTGC<br>TGATGAACCATTGCCTGCCGCTAAAGATTTGACTACAGCAGCCATATCTGGTCCAGGTATCTT<br>GTTAACACAAATGGCTGCACAAAGAGCTTTGAAGAGACAAGGTTTAGATTTGGACGCACACC<br>CACCTGTTGCAATTGCCGGTCATTCACAAGGTGTAACTGCTGTCGAATCCTTAAAAGCCGGTG<br>GTGCTAGAGATGTTGAATTGTTAGCAATAGGTCAATTGATCGGTGCCGCTGGTTCCTTAGTTA<br>GTAGAAGATGTGGTATGGTTGGTAGAGGTGACAGATCCCCTATGGTTAGTGTATTGAATGTT<br>GACCCAGCAAGAATGGCCGAATTGTTAGACGAATTTGCTCAAGATGTTAGAACTGTATTGCCA<br>CCTGCATTATCAATCAGAAACGGTAGAAGATCCGTTGTAATTACTGGTACACCAGAACAATTA<br>GCTAGATTCGAATTGTATTGCGAAAAGATTACCGAAAAGGAAGAAGCTGAAAGAAAGAATAA<br>GACTAGAGGTGGTGCAATTTTTAGACCAATATTCAATCAATTGAACGTCGAAGTTGGTTTTCA<br>TACACCTAGATTGGCTGGTGGTATTGATTTGGTTAACGAATGGGCAGCCAGAACTGGTTTGG<br>ATAGAGAATTAACTAGAGAATTGACCGAAACTATCTTCGTCAAGCCAGTTGATTGGGTATCAG<br>AAGTCGAAGGTTTGGCTGCTGCTGGTGCTAAATGGATTGTTGACTTAGGTCCATCCGATACAG<br>TCACCAGATTGACTGCACCTGTTATAAGAGGTTTAGGTATTGGTATAGTTGCTGCTGCTACAA<br>GAGCTGGTCAAAGATCATTGTTTACTGTAGGTGCAGAACCAGCTGTCGCACCTGCCTGGTCTT<br>CTTATGCTCCAAAGCCTGTAGCCTTGCCAGATGGTTCTGTCAAAGCATCTACTAAGTTCACTAG<br>ATTAACAGGTAGATCACCTATCTTGTTAGCTGGTATGACACCAACCACTGTCGATGCTAAAATT<br>GTTGCTGCTGCTGCTAATGCAGGTCACTGGGCTGAATTGGCTGGTGGTGGTCAAGTTACCGA<br>AGAAATTTTTGATGCCAGAATCGCTGAATTAACTCAATTGTTAGAACCAGGTAGAGCTGTTCA<br>ATTCAATAGTTTGTTTTTAGATCCATACTTGTGGAAGTTACAATTGGGTGGTAAAAGATTAGTT<br>CAAAAGGCTAGACAATCTGGTGCACCAATAGATGGTGTCGTTGTAACTGCTGGTATCCCTGAC<br>TTAGAAGAAGCAGTTGATTTGATCGAAGAATTGCATACAGTAGGTATATCAAACGTCGTTTTT<br>AAACCAGGTACTGTTGATCAAATTAAATCCGTAATCAAGATTGCAGCCGAAGTTCCTGGTAGA<br>GATGTAATAGTCCACGTTGAAGGTGGTAGAGCTGGTGGTCATCACTCTTGGGAAGATTTGGA<br>TGACTTGTTATTGTCTACTTATGGTGACTTGAGAAAGCATTCCAACATAACTATCTGTGTTGGT<br>GGTGGTATAGGTACACCAGAAAGAGCTGCTGAATATTTGTCTGGTGACTGGGCCAAAACTTA<br>CGGTTTTCCAGCTATGCCTGTAGATGGTATCTTGGTCGGTACTGCTGCTATGGCTACTTTAGAA<br>GCCACAACCTCTCCAGCTGTTAAGCAATTATTGGTAGACACTACAGGTACTGATACATGGGTT<br>GGTGCTGGTAAAGCCATTTCTGGTATGGCTTCTGGTAGATCACAATTAGGTGCAGACATCCAT<br>GAAATTGATAATACTGCTTCCAGATGCGGTAGATTATTGGATGAAGTTGCAGGTGACGCTGA<br>CGCAGTAGCCGAAAGAAGAGACGAAATTATAGCAGCTATGGCTGATACTGCAAAACCATACT<br>TTGGTGACGTCGCTGATATGACATATTTGCAATGGTTACAAAGATACGTTGAATTGGCTATTG<br>GTGACGGTGACAGTACCGCAGATACTGCTGCACCAGGTTCTCCTTGGTTAGCTGATACATGGA<br>GAGAAAGATTTGAAGAAATGTTGACCAGAGCTGAAGCAAGATTACATGAAAAGGATAGTGG<br>TCCTATCGAATCTTTGTTCGCCGCTGGTCCAGAATCACAAGCATTGTTGGATAATCCTGATACA<br>GCAATTGCAGCCTTATTGGCCAGATATCCAGATGCTGAAACAGTTAAATTGCACCCAGCTGAC<br>GTCCCATTTTTCGTTACCTTATGTAAAAAGCCAGGTAAACCTGTAAACTTTGTACCAGTCATTG<br>ATAAGGACGTCAGAAGATGGTGGAGATCAGATTCATTATGGCAAGCCCATGACGCTAGATAC<br>ACTGCTGATCAAGTCTGCATCATTCCAGGTACACAAGCCGTTGCTGGTATTACCAGAGTAGAC<br>GAACCTGTCGGTGAATTATTGGATAGATTCGAACAAGAAATCGTTGACAGAATTTTGGCAACA

GGTGCCGAACCAGTACCTGTAGTCAGTAGAAGACAAGCAAGAGCCGATGTAGCTGGTCCTTT
AGCAGTTGTATTGGACTCTCCAGATGTTTTATGGGCCGGTAGAACTGCTGTTAATCCTGTACA
CAGAATTGGTGCTCCAGGCGAATGGCAAGTCAATGATGTTCCAGGTAAACCTTCTGCAACACA
TCCAAACACCGGTGCAAGATTAGAATTGGCCGGTGACGGTTCCGTTACCTTAAGTGTACCTTT
GTCTGACATTTGGATAGATATCAGATTCACTTTGCCAGCAACCACTGTTGATGGTGGTGCCCC
TATCGTTACAGTAGAAGACGCTTCAACCGCAATGAGAGCCGTCTTGGCTATTGCTGCTGGTGT
TGACGGTCCAGATGCATTACCTGCCGTTGAAAATTCAACCGCTACTGTCTCCGTTGAATGGGA
CCCAGAAAAGGTCGCTGATCATACAGGTGTTACAGCAACCTTTGGTGCTCCATTGGCACCTGG
TTTAACCTTGGTCCCAGATGCTTTGGTTGGTTTATGTTGGCCAGCTGTTTTTGCCGCTGTTGGT
TCAGCTTTAACTGATGACGGTTTCCCAGTCGTTGAAGGTTTATTGTCCTTGGTTCATTTGGATC
ACGCAGCCCATTTGTTGGCAACTATGCCTGCCACAAAATCTGAATTGACCGTTTCAGCTACTGC
ATCTGCTGCTGTTGATACAGAAGTCGGTAGAGTAGTCCCAGTAGACGTCACCATCGCTGATTC
TGAAGGTACAGTTTTGGCCACCTTAGGTGAAAGATTTGCTATTAGAGGTAGAACTGGTGCAG
TTGAATTAACAGATCCACCTAGAGCCGGTGGTGCTATTACAGACAATGCTACTGATACTCCAA
GAAGAAGAAGAAGAGATGTTGTAGTCAACGCACCTGTTGACATGTCAGCCTTCGCTGTTGTAT
CCGGTGACCACAATCCAATACATACTGATAGAGCCGCTGCATTATTGGCTGGTTTGAAAATGC
CTATCGTTCACGGTATGTGGTTATCTGCCGCTGCACAACATGCAGTTACCGCCACTGATGGTA
GAGCTACCCCACCTGCAAGATTGGTTGGTTGGACTTCAAGATTTTTGGGTATGGTATTGCCAG
GTGACGAAATTGAGTTTAGAGTAGACAGAGTTGGTATCGATAGAGGTGCTGAAATTGTTGAA
GTAGCCGCTAAAGTAGGTGGTGAATTGGTCATGTCTGCAACTGCCCAATTAGCAGCCCCTAAA
ACAGTTTATGCTTTTCCAGGTCAAGGTATTCAATCAAAGGGTATGGGTATGGATGTCAGAGCA
AGATCCAAGGCTGCAAGAGCTGTTTGGGACACAGCAGATAAGTTTACTAGAGAAACCTTAGG
TTTCTCTGTTTTGCACGTCGTTAGAGATAACCCAACTAGTTTAATAGCTTCTGGTGTCCATTATC
ATCACCCTGAAGGTGTTTTATACTTGACTCAATTCACTCAAGTTGCTATGGCAACAGTAGCCGC
TGCACAAGTCGCCGAAATGAGAGAACAAGGTGCTTTCGTAGAAGGTGCCATTGCTTGTGGTC
ATAGTGTCGGTGAATATACTGCATTGGCCTGCGTTTCTGGTGTATACGAATTACCAGCTTTATT
GGAAGTAGTCTTTCACAGAGGTTCTAAGATGCATGACATTGTTCCAAGAGATGCTCAAGGTA
GATCAAATTATAGATTGGCCGCTATCAGACCTTCCCAAATTGATTTGGATGACGCAGACGTTA
AAGATTTCATAGCCGAAATCAGTGATAGAACTGGTGAATTTTTGGAAATCGTTAACTTCAACT
TAAGAGGTTCTCAATACGCTATCGCAGGTACTGTTGCTGGTTTGGAAGCATTGGAAGCAGAA
GTAGAAAAGAGAAGAGAATTGTCTGGTGGTAAAAGATCCTTTATTTTAGTTCCAGGTATCGAT
GTACCTTTCCATTCCAGTGTTTTGAGAGTCGGTGTTGCCGATTTTAGAAGAGCTTTAGAAAGA
GTTATGCCAGATCCTAATGATCCAGACTTATTAGTAGGTAGATATATTCCTAACTTGGTCCCAA
GACCTTTTACATTAGATAGAGACTTCGTACAAGAAATTAGAGATTTGGTCCCAGCAGAACCTT
TAGACGAAATATTGGCTGATTACGACACTTGGAGAAACGAAAGACCAAACGAATTATGTAGA
AAATTGGTAATAGAATTATTGGCCTGGCAATTTGCTTCTCCTGTCAGATGGATCGAAACTCAA
GATTTGTTGTTTATCGAAGAAGCAGCCGGTGGTTTGGGTGTTGAAAGATTCGTAGAAATTGG
TGTCAAGAATGCACCAACCGTTGCCGGTTTAGCTGCAAACACTTTGAAATTGCCTGAATACTCT
CATAACACAACCGAAGTATTGAACGCTGAAAGAGATGCCGCTGTCTTATTTGCAACAGATACC
GACCCAGAACCTGATTTGGACGAACCAGCTGGTGACTCTGCACCAGCTGCTGAAGCTGCACC
AGCCGAAGCTGTTCCAGCCGCTCCAGCTGCTCCAGCTCCAGCTGCTGCTCCATCAGGTGGTCC
AAGACCTGATGACATTACTTTCGACGCCAGTGATGCTACAATGGCATTGATCGCCTTGTCTGC
TAAGATCAGAATCGACCAAATCGAACCATTAGATTCCATAGAAAGTATAACTGATGGTGCTTC
TTCAAGAAGAAACCAAATGTTGGTTGATTTGGGTTCAGAATTAAACTTGGGTGCAATTGACGG
TGCTGCTGAAGCAGATTTGGCCGGTTTAAAAGCACAAGTTACAAAGTTGGCCAGAACCTATA
AACCATTTGGTCCTGTTTTAAGTGACGCTATAAATGATCAAGTCAGAACAGTTTTCGGTCCATC
TGGTAAAAGACCTGCATACATCGGTGAAAGAGTCAAAAAGACTTGGGAATTGGGTGAAGGTT
GGGTTAAACACGTAACCGTCGAAGTTGCTTTAGGTACTAGAGAAGGTTCCAGTGTTAGAGGT

GGTGCATTGGGTGGTTTACATGACGGTGCATTAGCCGATGCTGCTGCTGTTGATAAAGCTATT
GATGCTGCTGTTTCTGCTGTTGCAGCCAGAAGAGGTGTAGCTGTCTCTTTGCCATCTGCTGCT
GGTGCCGCTGGTGGTGTTGTAGATAGTGCAGCCTTGGGTGAATTTGCTGAACAAGTAACCGG
TCCTGATGGTGTCTTGGCATCTGCTGCAAGAACTATATTAGATCAATTGGGTTTGGGTGCTCC
ATTCGTTACACCTGACGCTAGTGCAGATGCCGAATTGATTGATTTGGTTACCGCAGAATTGGG
TTCTGACTGGCCAAGATTAGTTGCACCTGTATTCGATGGTAGAAAGGCTGTTTTATTGGATGA
CAGATGGGCTTCAGCTAGAGAAGATTTGGTTAGAATTTGGTTGATGGACGAAGGTGACATAG
AAGCTGATTGGGTTAGATTGTCCGAAAGATTCGAAGGTGCAGGTCATGTCGTTGGTACTCAA
GCCACTTATTGGCAGGGTAAAGCATTGGCCGCTGGTAGAAATGTTCACGCCTCTTTATATGCT
AGAGCAGCCGCTGGTGCAGAAAACCCAGCCACTGGTAGATACCATGATGAAGTTGCAGTAGT
CACAGGTGCCTCCAAAGGTAGTATTGCAGCCTCAGTTGTAGCTCAATTATTGGATGGTGGTGC
TACTGTCGTTGCAACTACATCCAAGTTAGATGACGCTAGATTGGCATTCTACAGAGAATTATA
CAGAGATAACGCCAGATTCGGTGCTAAATTGTGGGTTTTACCAGCCAATATGGCTTCATACAA
CGATATTGACGCTTTGGTCGAATGGGTTGGTACAGAACAAACCGAATCATTAGGTCCAAAATC
CATACATTTGAAGGATGCTTTGACTCCTACATTATTGTTTCCATTCGCTGCACCTAGAGTTGGT
GGTGACTTGTCAGATGCTGGTTCCAGAAGTGAAATGGAAATGAAAGTCTTATTGTGGGCAGT
TCAAAGATTAATAGGTGGTTTGTCTCACATCTCTTCAGATAGAGACATTGCCGCTAGATTACAT
GTAGTCTTGCCAGGTTCACCTAATAGAGGCATGTTTGGTGGTGACGGTGCTTATGGTGAATCC
AAGGCAGCCTTAGATGCAGTTGTAGCCAGATGGAAAGCTGAAACAAGTTGGGCACAAAGAG
TTTCTTTAGCCCACGCTTTGATAGGTTGGACTAGAGGTACAGGTTTGATGGGTCATAACGACG
TTATCGTAGATGCAGTAGAAGAAGCCGGTGTCACCACTTACTCAACTGAACAAATGGCTTCCA
TGTTATTGGACTTATGTGATGTTGAAACTAAGGTAGCTGCTGCTAGAGAACCAGTTCAAGCTG
ATTTGACAGGTGGTTTGGCAGAAGCCGAATTAGATTTGAGTGCTTTGGCAACAAAAGCTAGA
GAAGATGCAACCTCTGCCCCAGCTGATGACGCTGAAGATGACGAAGCTGCTGATCATTTGATT
GCCGCTTTGCCATCACCACCTAGACCTGCTGTTACAGCACCAGCCCCTGAATGGGCAGATTTG
GACGTTGATCCAGCCGATATGGTCGTTATAGTAGGTGGTGCTGAATTAGGTCCTTATGGTTCC
AGTAGAACTAGATACGAAATGGAAGTCGATAATGAATTGTCTGCTGCTGGTGTTTTAGAATTG
GCTTGGACAACCGGTTTGATTAAATGGGAAGATGACCCAAGACCTGGTTGGTATGACTCTGA
TTCTGGTGAATTGATAGATGAATCAGAAATCGTTGAAAGATACCACGACGAAGTAGTCTCCA
GATGCGGTATTAGAGAATTTGTTGATGACGGTGCTATAGACCCAGATCATGCAAGTCCTTTAT
TGGTCTCTGTTTTCTTAGACAAGGATTTTAGATTCGTTGTATCTTCAGAAGCTGATGCAAGAGC
CTTTGTTGAATTTGACCCAGAACATACAGCTGCAAGACCAATCCCTGACTCTGGTGACTGGGA
AGTCATAAGAAAAGCTGGTACTGAAATCAGAGTTCCAAGAAAAACTAAGTTGTCCAGAACAG
TAGGTGCACAAATTCCAACCGGTTTTGATCCTACTGTTTGGGGTATCAGTCCTGACATGGCTA
CTTCTATTGATAGAGTTGCATTGTGGAATATAGTAGCCACAGTCGATGCATTTTTGTCTTCTGG
TTTCACCCCAACTGAATTATTGAGATGGGTTCACCCTTCACAAGTAGCTTCCACCCAAGGTACT
GGTATGGGTGGTATGACTAGTATGCAAACAATGTATCATGGTAATTTGTTGGGTAGATCAAA
GCCAAACGATATCTTACAAGAAGTTTTGCCTAACGTCGTTGCCGCTCACGTCATGCAAAGTTAT
GTTGGTGGTTACGGTGCTATGGTTCATCCAGTAGCAGCCTGTGCTACTGTAGCAGTCTCTGTT
GAAGAAGGTGTTGATAAGATCAGATTGGGTAAAGCCGAATTTGTAGTCGCTGGTGGTTTCGA
TGACTTAACCTTGGAAGCTATAATCGGTTTTGGTGACATGGCTGCAACTGCCGATACAGAAAC
CATGAGAGCAAAAGGTATCTCCGATTCAAGATTTTCAAGAGCTAATGACAGAAGAAGATTGG
GTTTCTTAGAAGCTCAAGGTGGTGGTACTATTTTATTGGCAAACGGTGCTTTAGCATTGCAAA
TGGGTTTGCCAGTTTTAGCTGTTGTAGGTTACGCACAAAGTTTTGCCGATGGTGTTCATACTTC
TATACCAGCACCTGGTTTGGGTGGTTTAGGTGCTGGCAGAGGTGGTAGAGAATCTCAATTGG
CTAGATCCTTGGCAAAGTTAGGTGTTGGTGCCGATGACATCGCTGTCGTTAGTAAACACGACA
CTTCTACATTAGCTAATGATCCAAACGAAACAGAATTACATGAAAGATTGGCCGATGCTTTAG
GTAGAGCACCAGGTAATCCTTTGTTCGTAGTCTCTCAAAAATCATTAACTGGTCACTCTAAGG

	GTGGTGCCGCTGCATTCCAATTGATGGGTTTATGTCAAATGTTAAGAGATGGTGTCATTCCAC<br>CTAACAGATCATTGGACTGCGTTGATGACGAATTAGCCACTTCCGCTCATTTTGTTTGGCCAAG<br>AGAAACATTAAGATTGGGTGACAAATATCCTTTGAAGGCTGGTTTAGTTACAAGTTTGGGTTT<br>TGGTCACGTTTCTGGTTTGATTGCCTTAGTACATCCACAAGCATTTTTGGCCGCTTTAGCACCT<br>GAACAAAGAGCAGCCTACACTGCACAAGCCCAAGAAAGAGCTTTGGCAGGTCAAAGAAGATT<br>AGCCTCTGCTATTGCAGGTGGTAGACCAATGTTTGAAAGACCTGCTGATAGAAGATTCGACG<br>GTGACCAACCAGAAAAGACTCAAGAAGCTGCTATGTTATTGGACCCTGCCTCAAGATTAGGT<br>GAAAATGGTGTTTACAGAAGATGA
AcpS	ATGTTGCCAGTCGGTATAGTTGGTATTGGTATTGATTTGGTTTCTATCCCAGAATTTGCAGAAC<br>AAGTAGACAGACCTGGTACTGTCTTTGCTGAAACTTTCACACCTGGTGAAAGAAGAGATGCTG<br>CTGATAAATCTTCATCCGCCGCTAGACATTTGGCAGCCAGATGGGCTGCAAAAGAAGCAGTA<br>ATTAAGGCCTGGAGTGGTTCAAGATTCGCCAAGAGACCAGTCTTACCTGAAGCTATCCATAGA<br>GATATCGAAGTTATTACAGACATGTGGGGTAGACCAAGAGTTAGATTGTCAGGTGCCGTAGC<br>TGAACACTTAAAGGAAGTTACCATCCATTTGTCCTTAACCCACGAAGCTGATACTGCTGCTGCT<br>GTTGCAGTATTGGAAGAAAGATAA
AtFAR	ATGGAATCTAATTGTGTTCAATTCTTGGGTAATAAGACTATCTTGATCACAGGTGCACCAGGTT<br>TCTTGGCTAAGGTTTTGGTTGAAAAGATTTTGAGATTGCAACCAAATGTTAAGAAAATATATTT<br>GTTGTTGAGAGCACCAGATGAAAAATCTGCTATGCAAAGATTGAGATCAGAAGTTATGGAAA<br>TTGATTTGTTTAAAGTTTTGAGAAACAATTTGGGTGAAGATAATTTGAACGCTTTGATGAGAG<br>AAAAGATTGTTCCAGTTCCAGGTGACATCTCTATCGATAATTTGGGTTTGAAGGATACTGATTT<br>GATCCAAAGAATGTGGTCAGAAATCGATATCATTATTAACATTGCTGCAACTACAAACTTCGA<br>TGAAAGATACGATATTGGTTTAGGTATTAATACATTCGGTGCATTAAACGTTTTGAACTTCGCT<br>AAGAAATGTGTTAAGGGTCAATTGTTGTTGCATGTTTCTACTGCTTACATTTCAGGTGAACAAC<br>CAGGTTTGTTATTGGAAAAGCCTTTTAAAATGGGTGAAACATTGTCTGGTGACAGAGAATTAG<br>ATATCAACATCGAACATGATTTGATGAAACAAAAATTGAAAGAATTGCAAGATTGTTCTGATG<br>AAGAAATTTCACAAACTATGAAGGATTTTGGTATGGCAAGAGCTAAATTGCATGGTTGGCCA<br>AACACTTATGTTTTTACAAAGGCAATGGGTGAAATGTTGATGGGTAAATACAGAGAAAATTTG<br>CCATTGGTTATTATCAGACCAACTATGATCACTTCTACAATTGCTGAACCATTTCCAGGTTGGA<br>TCGAAGGTTTGAAGACATTGGATTCAGTTATTGTTGCTTATGGTAAAGGTAGATTGAAGTGTT<br>TCTTGGCTGATTCTAATTCAGTTTTCGATTTGATCCCAGCAGATATGGTTGTTAATGCTATGGT<br>TGCTGCTGCTACTGCACATTCAGGTGACACAGGTATTCAAGCTATCTATCATGTTGGTTCTTCA<br>TGCAAGAACCCAGTTACTTTCGGTCAATTGCATGATTTCACAGCAAGATACTTCGCTAAAAGA<br>CCATTAATCGGTAGAAACGGTTCTCCAATCATCGTTGTTAAGGGTACTATCTTATCTACAATGG<br>CACAATTTTCATTGTACATGACATTGAGATACAAATTGCCATTGCAAATTTTAAGATTGATTAA<br>TATCGTTTACCCTTGGTCTCATGGTGACAACTACTCTGATTTGTCAAGAAAAATTAAATTGGCT<br>ATGAGATTGGTTGAATTGTACCAACCATACTTATTGTTTAAAGGTATTTTTGATGATTTGAATA<br>CTGAAAGATTGAGAATGAAGAGAAAGGAAAACATCAAAGAATTGGATGGTTCTTTTGAATTT<br>GATCCAAAGTCAATCGATTGGGATAACTACATCACTAACACACATATCCCAGGTTTGATCACA<br>CATGTTTTGAAACAATAA
AmFAR	ATGTCAACTATTTCCGATAATCAATGCACTTCCGTCAGAGACTTTTACAAGGACAGATCCATAT<br>TCATCACTGGTGGTACAGGTTTCATGGGTAAAGTCTTGGTAGAAAAGTTGTTAAGATCCTGTC<br>CAGGTATTAAAAATATCTATATCTTGATGAGACCTAAAAAGAGTCAAGATATACAACAAAGAT<br>TGCAAAAGTTGTTGGATGTTCCATTGTTCGACAAGTTGAGAAGAGATACACCAGACGAATTGT<br>TGAAGATCATCCCTATTGCTGGTGACGTTACCGAACATGAATTAGGTATCTCTGAAGCTGATC<br>AAAATGTCATCATCAGAGACGTATCTATCGTTTTCCATTCAGCTGCAACTGTAAAGTTCGATGA<br>ACCTTTGAAAAGATCTGTTCACATCAACATGATTGGTACAAAGCAATTGTTGAATTTGTGTCAT<br>AGAATGCACAACTTAGAAGCCTTGATTCACGTTTCAACCGCTTATTGTAATTGCGATAGATAC

GACGTCGCAGAAGAAATCTATCCAGTATCTGCAGAACCTGAAGAAATCATGGCCTTGACTAA
GTTGATGGATTCACAAATGATCGACAATATCACTCCAACATTGATTGGTAATAGACCTAACAC
CTACACTTTTACAAAGGCATTGACAGAAAGAATGTTGCAATCCGAATGCGGTCATTTGCCAAT
CGCTATTGTAAGACCTTCCATTGTTTTATCTTCATTCAGAGAACCAGTTAGTGGTTGGGTCGAT
AATTTGAACGGTCCTACAGGTATTGTTGCCGCTGCAGGTAAAGGTTTCTTTAGATCTATGTTGT
GTCAAAAGAATATGGTTGCTGATTTGGTTCCAGTCGACATCGTTATTAATTTGATGATCTGCAC
CGCTTGGAGAACCGCAACTAACAGAACAAAAACCATTCCAATCTATCATTGTTGCACTGGTCA
ACAAAATCCTATTACATGGCAACAATTCGTTGAATTAATCTTGAAGTACAACAGAATGCATCC
ACCTAACGATACCATTTGGTGGCCAGACGGTAAATGTCACACTTTCGCAATCGTCAACAACGT
ATGCAAGTTGTTCCAACATTTGTTACCAGCCCACATCTTGGATTTCATTTTCAGATTGAGAGGT
AAACCTGCTATCATGGTCGGTTTGCACGAAAAGATTGACAAGGCAGTAAAGTGTTTGGAATA
CTTCACTATGCAACAATGGAATTTCAGAGATGACAACGTTAGACAATTATCCGGTGAATTGAG
TCCAGAAGATAGACAAATTTTTATGTTCGATGTTAAGCAAATCGACTGGCCTTCTTATTTGGAA
CAATACATCTTGGGTATAAGACAATTCATTATTAAAGATTCTCCAGAAACATTGCCTGCCGCTA
GATCACATATTAAGAAATTGTACTGGATTCAAAAGGTTGTCGAATTCGGCATGTTGTTAGTAG
TTTTGAGATTCTTGTTGTTGAGAATTCCAATGGCACAATCCGCCTGTTTCACTTTGTTGAGTGC
TATATTAAGAATGTGCAGAATGATAGTTTAA

CfFAR

ATGGAAGTTAACAACAACTCACCAGTTGTTCAATTCTACGATAATAAGACAGTTTTTATTACAG
GTGCTACTGGTTTTATGGGTAAAGTTTTGGTTGAAAAATTGTTAAGATCAACTAACATCTTGAA
ATTGTTGTTGTTGATCAGACCAAAGAAAGGTGTTCAAACAGAACAAAGATTGCAAACTTTGTT
ATCTTCATCTGTTTTCGATAGAGTTAGAGAAATCGATCCAGCTTTGTTAGAAAAGGTTGAAGTT
GTTAACGGTGACATCACAGAAGATAATTTGGGTATCGATGAAGAAGCAGAAAGAATTTTAAC
AGAATCAGTTAACGTTGTTTTTCATTGTGCTGCAACAGTTAGATTCGATGAAGATTTGACTAAG
TCAGTTGCTATGAACGTTTCTGCTGTTTTGGCAATCATTGATTTGGCTAAGAAAACTAAGAAAT
TGGAAGCATTGGTTGATGTTTCAACTGCATACTGTAATTGTGATTTGAAAAATATCGATGAAA
TCATATATCCACCACCAGGTAATCCAAGAGGTTTGGTTGATTGTTGTAAGTGGATGGATTCTG
AAAAATTGGATGGTCCAGAAATGACAAAAATTATGATTGGTAACAGACCAAACACTTACACTT
TTACTAAAGCATTGGCAGAAGCTGTTTTACATACAGAAGGTGCTTCATTGCCATTGGCAATCA
TCAGACCATCTATCGTTACTGCTGCATGGAAAGAACCATTTCCAGGTTGGGTTGATAATTTTAA
TGGTGCTACAGGTGTTTTGGCAGGTGCTGGTGCAGGTTTGATGAGAACTTTGTACTGTAAGA
GATCATGTGTTGCAGATATGGTTCCAGTTGATGTTTGTATCAATTTGATGTGTGTTTTGGGTTG
GAAAGCTGCATCACAACCAGCTTCTACTACACCAGTTTACAACTGTACATCTGGTGGTATTAAT
CCAATTACTTGGGGTCAAGTTGAAGCATGGGGTTTGCAAACATTGGTTGATAACCCATACCAA
GATGTTTTCTGGTATCCAGGTGGTTCTTACAAAGAAAATTGGTATTTGAACAGATTTTTCCAAT
TGTTGTTCCATTATGGTCCAGCTCATTGTGTTGATTTGTTATGTAGATTGTCTGGTAGAAAGCC
ATTTTTAGTTAAAATTTCAAACATGATGCAAAAATCTACTAAAGCATTGGAACCTTTTACTACA
AATTCATGGAACTGGTCTAACAACAACGTTGTTAAATTGGAAGGCGAATTAACAGTTGAAGAT
AGAGCTGTTTTCGGTTTCGATATCAAGACTGATTTCGATTGGAAGGAATATTTGGCAATCTAT
GTTCAAGGTATTAGAAAGTTCTTGTTTAAATCAGATCCATCTACTTTACCAGGTTCAAGAAGAT
ACATGTAA

ScFAR

ATGGAAGAAATGGGTTCAATCTTGGAATTCTTGGACAATAAGGCTATCTTGGTCACAGGTGCA
ACAGGTAGTTTGGCTAAAATCTTTGTTGAAAAAGTCTTGAGATCACAACCTAACGTTAAAAAG
TTGTATTTGTTGTTGAGAGCTACCGATGACGAAACTGCTGCATTGAGATTGCAAAACGAAGTT
TTCGGTAAAGAATTGTTTAAAGTCTTGAAGCAAAATTTGGGTGCAAACTTTTACTCTTTCGTAT
CAGAAAAGGTTACCGTTGTCCCAGGTGACATAACTGGTGAAGACTTGTGTTTGAAGGATGTT
AATTTGAAGGAAGAAATGTGGAGAGAAATTGATGTAGTTGTCAACTTAGCCGCTACCATTAAT
TTCATCGAAAGATACGACGTATCCTTGTTGATTAACACTTACGGTGCAAAGTACGTTTTGGATT
TCGCCAAAAAGTGCAATAAGTTGAAGATCTTCGTCCATGTATCTACTGCTTACGTTTCAGGTGA

	AAAGAATGGTTTGATCTTGGAAAAGCCTTATTACATGGGTGAATCTTTGAACGGTAGATTGGG<br>TTTGGATATCAACGTCGAAAAGAAATTGGTTGAAGCCAAGATTAATGAATTGCAAGCAGCCG<br>GTGCTACAGAAAAATCCATTAAGAGTACCATGAAGGATATGGGTATAGAAAGAGCAAGACAC<br>TGGGGTTGGCCAAATGTTTATGTCTTTACCAAAGCCTTGGGTGAAATGTTATTGATGCAATAC<br>AAGGGTGACATTCCTTTGACAATAATCAGACCAACCATCATCACTTCTACATTCAAAGAACCAT<br>TCCCTGGTTGGGTAGAAGGTGTTAGAACAATAGATAACGTACCTGTTTACTACGGTAAAGGTA<br>GATTGAGATGTATGTTATGCGGTCCTTCAACTATAATCGACTTAATCCCAGCTGATATGGTAGT<br>TAACGCTACAATTGTCGCAATGGTAGCACATGCCAATCAAAGATATGTTGAACCAGTCACTTA<br>CCACGTTGGTTCTTCAGCTGCAAATCCTATGAAATTATCCGCCTTGCCAGAAATGGCTCATAGA<br>TACTTCACAAAGAATCCATGGATAAACCCTGATAGAAATCCAGTCCATGTAGGTAGAGCTATG<br>GTCTTTTCCAGTTTCAGTACTTTCCACTTGTATTTGACATTGAACTTTTTATTGCCATTGAAGGT<br>TTTGGAAATCGCAAACACAATTTTCTGTCAATGGTTCAAGGGTAAATACATGGACTTGAAGAG<br>AAAGACCAGATTGTTGTTGAGATTGGTTGATATCTATAAACCTTACTTATTTTTCCAAGGTATC<br>TTCGATGACATGAACACAGAAAAGTTAAGAATAGCCGCTAAGGAATCTATCGTTGAAGCTGA<br>CATGTTTTATTTCGATCCAAGAGCAATTAACTGGGAAGATTATTTCTTAAAAACTCACTTTCCT<br>GGTGTCGTTGAACATGTATTGAACTAA
TaFAR	ATGGTTGATACATTGTCTGAAGAAAACATAATTGGTTACTTTAAAAATAAGTCCATCTTGATTA<br>CTGGTTCCACAGGTTTCTTGGGCAAGATTTTGGTTGAAAAGATATTGAGAGTACAACCAGATG<br>TTAAGAAAATATATTTGCCTGTCAGAGCAGTAGACGCTGCAGCCGCTAAACATAGAGTTGAA<br>ACTGAAGTTGTCGGTAAAGAATTGTTCGGTTTGTTGAGAGAAAAGCACGGTGGTAGATTCCA<br>ATCATTCATATGGGAAAAGATCGTCCCATTAGCTGGTGACGTAATGAGAGAAGATTTCGGTGT<br>AGACTCTGAAACATTAAGAGAATTGAGAGTTACCCAAGAATTAGATGTTATTGTCAATGGTGC<br>AGCCACTACAAACTTTTATGAAAGATACGATGTCGCATTGGACGTAAACGTTATGGGTGTAAA<br>GCATATGTGTAACTTCGCCAAAAAGTGCCCAAACTTGAAGGTCTTGTTACACGTATCTACCGC<br>ATATGTAGCCGGTGAAAAACAAGGTTTAGTTCAAGAAAGACCTTTTAAGAATGGTGAAACCTT<br>GTTAGAAGGTACTAGATTGGATATAGACACAGAATTGAAGTTGGCTAAGGATTTGAAAAAGC<br>AATTAGAAGCAGATGTTGACTCTTCACCAAAAGCTGAAAGAAAAGCAATGAAGGACTTAGGT<br>TTGACAAGAGCAAGACATTTTAGATGGCCTAATACTTACGTTTTCACAAAGAGTATGGGTGAA<br>ATGGTCTTATCTCAATTGCAATGTGATGTACCAGTAGTTATAGTTAGACCTAGTATCATCACTT<br>CTGTTCAAAACGATCCATTGCCTGGTTGGATCGAAGGTACCAGAACTATTGACACAATCGTTA<br>TCGGTTACGCCAAGCAAAATTTGACTTACTTTTTAGCTGATTTGAACTTGACAATGGATGTCAT<br>GCCAGGTGACATGGTCGTAAATGCCATGATGGCTGCAATCGTTGCTCATTCCAGTTCTTCATT<br>AGAAAAGACTAAGTCACATCCAAAGCAACACGCCCCTGCTGTTTACCACGTCTCCAGTTCTTTG<br>AGAAATCCAGCACCTTATAACGTTTTACATGAAGCCGGTTTTAGATACTTCACAGAACACCCA<br>AGAGTTGGTCCTGATGGTAGAACAGTCAGAACCCATAAAATGACCTTTTTGTCATCCATGGCT<br>TCATTCCACTTGTTCATGATGTTGAGATACAGATTGTTGTTGGAATTGTTGCATTTGTTGTCCAT<br>CTTGTGTTGCGGTTTGTTCGGTTTGGATACATTGTACCATGACCAAGCTAGAAAGTACAGATT<br>CGTTATGCACTTGGTTGATTTGTACGGTCCATTTGCATTGTTCAAAGGTTGCTTCGATGACGTT<br>AATTTGAACAAGTTGAGATTGGCTATGACCTCAAATCATGGTTCCTTGTTTAACTTCGATCCTA<br>AAACTATAGATTGGGACGAATACTTCTACAGAGTCCACATACCAGGTGTCATAAAATACATGT<br>TGAAATAA

Supplementary Table 7. Plasmids used in this study.

Plasmids	Genotype or characteristics	Base plasmid	Source
pSPGM2	pSPGM2	pSPGM2	R&D systems
pELO2	pSPGM2-ELO2	pSPGM2	This work
pELO3	pRS316-ELO3	pRS316	Ref[8]
p415GPD	p415GPD	p415GPD	R&D systems
pGPD415-MvFAS-AcpS	pGPD415-MvFAS-AcpS	p415GPD	This work
pSPGM2-MvFAS-AcpS	pSPGM2-MvFAS-AcpS	pSPGM2	This work
pVLCFAlc01	pSPGM2-AmFAR	pSPGM2	This work
pVLCFAlc02	pSPGM2-AtFAR	pSPGM2	This work
pVLCFAlc03	pSPGM2-CfFAR	pSPGM2	This work
pVLCFAlc04	pSPGM2-ScFAR	pSPGM2	This work
pVLCFAlc05	pSPGM2-TaFAR	pSPGM2	This work
pVLCFAlc06	pSPGM2-AmFAR-Elo2	pSPGM2-ELO2	This work
pVLCFAlc07	pSPGM2-AtFAR-Elo2	pSPGM2-ELO2	This work
pVLCFAlc08	pSPGM2-CfFAR-Elo2	pSPGM2-ELO2	This work
pVLCFAlc09	pSPGM2-ScFAR-Elo2	pSPGM2-ELO2	This work
pVLCFAlc10	pSPGM2-TaFAR-Elo2	pSPGM2-ELO2	This work
p416GPD	p416GPD	p416GPD	R&D systems
pDynCon01	p416TDH3p-AtFAR	p416GPD	This work
pDynCon02	p416TEF1p-AtFAR	p416GPD	This work
pDynCon03	p416GAL1p-AtFAR	p416GPD	This work
pDynCon04	p416HXT7p-AtFAR	p416GPD	This work
pDynCon05	p416ADH2p-AtFAR	p416GPD	This work
pDynCon06	p416ICL1p-AtFAR	p416GPD	This work
pDynCon07	p416-ELO3-HXT1p-TDH3p-AtFAR	p416GPD	This work
pDynCon08	p416-ELO3-HXT1p-TEF1p-AtFAR	p416GPD	This work
pDynCon09	p416-ELO3-HXT1p-GAL1p-AtFAR	p416GPD	This work
pDynCon10	p416-ELO3-HXT1p-HXT7p-AtFAR	p416GPD	This work
pDynCon11	p416-ELO3-HXT1p-ADH2p-AtFAR	p416GPD	This work
pDynCon12	p416-ELO3-HXT1p-ICL1p-AtFAR	p416GPD	This work
pROS10	pROS10	pROS10	Ref[7]
pROS10-gRNA:elo3	pROS10-gRNA:elo3	pROS10	This work
pROS13	pROS13	pROS13	Ref[7]
pROS13-gRNA:gal1	pROS13-gRNA:gal1	pROS13	This work

Supplementary Table 8 Overview Fatty Acyl Reductase (FARs).

Gene	Species	NCBI accession	Primary substrate specificity	Reference
AmFAR	*Apis mellifera*	CAD67815	C22:0 C24:0 C26:0	Ref[9]
AtFAR	*Arabidopsis thaliana*	NP_197642.1	C22:0	Ref[9]
CfFAR	*Calanus finmarchicus*	AEO89345.1	C20:0 C22:0 C24:0 C26:0	Ref[10]
ScFAR	*Simmondsia chinensis*	AAD38039	C20:0 C22:1	Ref[9]
TaFAR	*Triticum aestivum*	CAD30692.1	C22:0	Ref[11]

Reference

1. Boehringer, D., Ban, N. & Leibundgut, M. 7.5-angstrom Cryo-EM Structure of the Mycobacterial Fatty Acid Synthase. *J Mol Biol* **425**, 841-849 (2013).
2. Kaneda, K., Imaizumi, S. & Yano, I. Distribution of C22-, C24- and C26-alpha-unit-containing mycolic acid homologues in mycobacteria. *Microbiol Immunol* **39**, 563-570 (1995).
3. Rainey, F.A. & Oren, A. Taxonomy of Prokaryotes - Introduction. *Method Microbiol* **38**, 1-5 (2011).
4. Oh, C.S., Toke, D.A., Mandala, S. & Martin, C.E. ELO2 and ELO3, homologues of the Saccharomyces cerevisiae ELO1 gene, function in fatty acid elongation and are required for sphingolipid formation. *J Biol Chem* **272**, 17376-17384 (1997).
5. Paul, S. et al. Members of the Arabidopsis FAE1-like3-ketoacyl-CoA synthase gene family substitute for the Elop proteins of Saccharomyces cerevisiae. *J Biol Chem* **281**, 9018-9029 (2006).
6. Valle-Rodriguez, J.O., Shi, S.B., Siewers, V. & Nielsen, J. Metabolic engineering of Saccharomyces cerevisiae for production of fatty acid ethyl esters, an advanced biofuel, by eliminating non-essential fatty acid utilization pathways. *Appl Energ* **115**, 226-232 (2014).
7. Mans, R. et al. CRISPR/Cas9: a molecular swiss army knife for simultaneous introduction of multiple genetic modifications in Saccharomyces cerevisiae. *Yeast* **32**, S253-S253 (2015).
8. Wenz, P., Schwank, S., Hoja, U. & Schuller, H.J. A downstream regulatory element located within the coding sequence mediates autoregulated expression of the yeast fatty acid synthase gene FAS2 by the FAS1 gene product. *Nucleic Acids Res* **29**, 4625-4632 (2001).
9. Rowland, O. & Domergue, F. Plant fatty acyl reductases: Enzymes generating fatty alcohols for protective layers with potential for industrial applications. *Plant Sci* **193**, 28-38 (2012).
10. Teerawanichpan, P. & Qiu, X. Molecular and Functional Analysis of Three Fatty Acyl-CoA Reductases with Distinct Substrate Specificities in Copepod Calanus finmarchicus. *Mar Biotechnol* **14**, 227-236 (2012).
11. Wang, Y. et al. Molecular Characterization of TaFAR1 Involved in Primary Alcohol Biosynthesis of Cuticular Wax in Hexaploid Wheat. *Plant Cell Physiol* **56**, 1944-1961 (2015).
